# Advancing Multi‐Ion Sensing with Poly‐Octylthiophene: 3D‐Printed Milker‐Implantable Microfluidic Device

**DOI:** 10.1002/advs.202408314

**Published:** 2024-10-14

**Authors:** Md. Azahar Ali, Matin Ataei Kachouei

**Affiliations:** ^1^ School of Animal Sciences Virginia Tech Blacksburg Virginia 24061 USA; ^2^ Biological Systems Engineering Virginia Tech Blacksburg Virginia 24061 USA

**Keywords:** additive manufacturing, implantable, ion‐to‐electron transducer, livestock, milking parlor, multi‐ion sensor, poly‐octylthiophene, subclinical hypocalcemia

## Abstract

On‐site rapid multi‐ion sensing accelerates early identification of environmental pollution, water quality, and disease biomarkers in both livestock and humans. This study introduces a pocket‐sized 3D‐printed sensor, manufactured using additive manufacturing, specifically designed for detecting iron (Fe^2+^), nitrate (NO_3_^–^), calcium (Ca^2+^), and phosphate (HPO_4_^2−^). A unique feature of this device is its utilization of a universal ion‐to‐electron transducing layer made from highly redox‐active poly‐octylthiophene (POT), enabling an all‐solid‐state electrode tailored to each ion of interest. Manufactured with an extrusion‐based 3D printer, the device features a periodic pattern of lateral layers (width = 80 µm), including surface wrinkles. The superhydrophobic nature of the POT prevents the accumulation of nonspecific ions at the interface between the gold and POT layers, ensuring exceptional sensor selectivity. Lithography‐free, 3D‐printed sensors achieve sensitivity down to 1 ppm of target ions in under a minute due to their 3D‐wrinkled surface geometry. Integrated seamlessly with a microfluidic system for sample temperature stabilization, the printed sensor resides within a robust, pocket‐sized 3D‐printed device. This innovation integrates with milking parlors for real‐time calcium detection, addressing diagnostic challenges in on‐site livestock health monitoring, and has the capability to monitor water quality, soil nutrients, and human diseases.

## Graphical abstract

This work introduces a pocket‐sized, milker‐attachable 3D‐printed sensing platform designed for rapid, on‐site detection of Fe^2+^, NO_3_^−^, Ca^2+^, and HPO_4_^2−^. Featuring a universal ion‐to‐electron transducing layer, the device provides exceptional selectivity and sensitivity down to 1 ppm. Its innovative 3D‐wrinkled surface, integrated with a microfluidic system, allows for real‐time monitoring of milk Ca^2+^ levels, facilitating on‐site livestock health management.

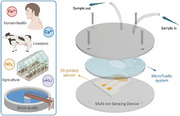

## 1 Introduction

Accurate, rapid, and continuous multi‐ion sensing in liquid mediums has many potential applications,^[^ ^1^ ^]^ including environmental monitoring,^[^ ^2^ ^]^ soil monitoring,^[^ ^3^ ^]^ animal diagnostics,^[^ ^4^ ^]^ plant monitoring,^[^ ^5^ ^]^ and genome sequencing.^[^ ^6^ ^]^ Recently, solid‐state‐based potentiometric sensors have gained interest compared to traditional advanced tools including spectrophotometry,^[^ ^7^ ^]^ chromatography,^[^ ^8^ ^]^ and mass spectrometry.^[^ ^9^ ^]^ Such sensors are miniaturized, easy to calibrate, simple to interface with electronics and enable on‐site field testing.^[^ ^10^ ^]^ There are two extensively used modalities of solid‐state sensors: ion‐selective electrochemical electrodes^[^ ^11^ ^]^ and ion‐selective field effect transistors (ISFETs),^[^ ^12^ ^]^ which incorporate the ion‐selective membranes containing specific ionophores. Such sensors work based on Nernst's law,^[^ ^13^ ^]^ where the ion of interest binds reversibly to the membrane, developing a potential in correlation to the logarithm of the ion concentration.^[^ ^14^ ^]^ The resulting potential difference, called Open Circuit Potential (OCP), enabled by such an ion exchange mechanism^[^ ^14^ ^]^ at equilibrium, is directly dependent on the sensor output.^[^ ^13^, ^14^ ^]^ OCP involves measuring the equilibrium potential difference between a working electrode and a reference electrode without any external current, providing information about the ion concentration in the surface and bulk solution. However, the reversible binding of nontarget ions can also influence this potential output, posing a major concern for sensor selectivity.^[^ ^15^ ^]^ Such nontarget ion binding can lead to signal drifting, necessitating sensor recalibration.^[^ ^16^ ^]^ Several other issues also exist with state‐of‐the‐art potentiometric sensors.^[^ ^16^ ^]^ For example, the delamination of ion‐selective membranes (ISMs),^[^ ^17^ ^]^ formation of a water layer beneath the ISM,^[^ ^18^ ^]^ and poor quality of reference electrodes^[^ ^19^ ^]^ limit the long‐time measurement of ions. The formation of a water layer between the ISM and electrode layer reduces sensor stability.^[^ ^20^ ^]^

To address these concerns, several electroactive materials including polyaniline,^[^ ^21^ ^]^ poly(3‐octylthiophene),^[^ ^22^ ^]^ poly(3,4‐ethylenedioxythiophene): poly(styrene‐sulfonate),^[^ ^23^ ^]^ and carbon layers^[^ ^24^ ^]^ have been investigated as all‐solid‐state ion‐to‐electron transducing layers. These materials, with their hydrophobicity and high redox properties, when combined with ISMs, reduce the potential drifting and improve the stability and selectivity of the sensors.^[^ ^25^ ^]^ However, limited surface areas of planar 2D geometries^[^ ^26^ ^]^ of these materials restrict the sensor performance in terms of sensitivity, stability, and long‐term field‐deployment,^[^ ^27^ ^]^ thereby limiting their effectiveness in practical applications requiring reliable and accurate ion sensing.

Additively manufactured (i.e., 3D‐printed) sensors are currently gaining significant attention due to their customizability, miniaturization, elasticity, and ability to create complex geometries with controlled structures.^[^ ^28^ ^]^ The layer‐by‐layer printing capability of multiple materials in 3D printing allows for manufacturing a fully functional device on demand.^[^ ^29^ ^]^ The unique features of lithography‐free manufacturing in 3D printing (without requiring cleanroom facilities) enable the creation of personalized, shaped 3D surfaces or scaffolds that seamlessly interface with other materials to produce functional devices.^[^ ^30^ ^]^ Each sensor can be fabricated in one to several minutes using inexpensive extrusion or other 3D printers (priced ≈ $2300), demonstrating cost‐effectiveness per test in developing areas.^[^ ^31^ ^]^ Due to their multi‐length‐scale sensing structures, 3D‐printed sensors with high surface areas can surpass the biomolecular limit‐of‐detection barrier.^[^ ^32^ ^]^ For instance, a free‐standing, 3D‐printed sensor with carbon microelectrodes was manufactured using two‐photon lithography followed by pyrolysis for detecting neurotransmitters such as dopamine.^[^ ^30^ ^]^ The high elasticity of the microelectrodes, combined with a carbon electroactive surface, enables implantability into real tissue.^[^ ^30^ ^]^ Such customization facilitates easy manufacturing with high sensor performance, including high sensitivity, low limit‐of‐detection, and field deployability.

On the other hand, the poly‐octylthiophene (POT) has been utilized as a solid‐contact (SC) layer in various sensing applications, including chloride (Cl^–^) ions,^[^ ^33^ ^]^ potassium ion (K^+^),^[^ ^34^ ^]^ drug (neostigmine) analyte,^[^ ^35^ ^]^ azides,^[^ ^36^ ^]^ and nitrate.^[^ ^36^ ^]^ The solid‐contact POT layer not only provides high reproducibility and redox activity but also maintains stable interfacial potentials between the potential collector (base electrode) and the POT layer, offering an excellent interface with ion‐selective membranes (ISMs) without forming a water layer.^[^ ^33^, ^37^ ^]^ This results in reduced potential drift, improved sensor stability and selectivity, reproducible interfacial potential, and miniaturization. Furthermore, the solution process for poly(3‐octylthiophene) allows highly uniform patterning on any 3D surface through a solution‐cast method.^[^ ^38^ ^]^ The solid‐contact POT transducing layer offers versatile applications, ideal for detecting multiple ions^[^ ^39^ ^]^ in complex liquid matrices and integrating them into microfluidic systems and milking parlors for lactating cows. Sensor integration with milkers enables online disease monitoring, promoting early treatment and prevention. Ultimately, POT serves as a universal transduction layer, excelling in sensing with stable potentials, high reproducibility, and versatile ion detection, integrating into complex systems for disease monitoring and economic benefits.

In this study, we demonstrate high‐resolution and continuous sensing of multiple ions using a universal 3D‐printed sensor featuring periodic patterns of POT layers with surface wrinkles. This sensor is characterized by its low cost, miniaturization, customizability, selectivity, and capability for long‐term ion measurements. The sensor comprises a simple geometry with two electrodes—an extrusion 3D‐printed working electrode and a reference electrode. The periodic layers (width ≈80 µm) and surface wrinkles are achieved through lateral layer‐by‐layer 3D printing, enabling sensitivity down to 1 ppm of target ions. Specific ISMs on these 3D‐printed sensors selectively measure ions such as iron (Fe^2+^), nitrate (NO_3_^–^), calcium (Ca^2+^), and phosphate (PO_4_^2–^). The periodic POT layer acts as a redox capacitive solid‐contact layer, facilitating ion‐to‐electron transduction through an ion‐exchange mechanism. This versatile 3D sensing platform finds practical applications in plant science (e.g., detecting Fe^2+^ in plants), soil and water science (monitoring NO_3_^−^ and PO_4_^2−^ levels), and animal science (measuring Ca^2+^ and HPO_4_^2−^ in lactating cows) for diagnostic and health evaluation purposes. For instance, in livestock monitoring, the sensor integrates with a PDMS‐based long‐channel microfluidic system, a peristaltic pump, and a compact 3D‐printed holder with a universal design. This configuration minimizes temperature fluctuations, thereby enhancing ion sensing accuracy. Its durability and portability enable seamless integration into milking parlors for direct monitoring of hypocalcemia (calcium ion tracking) in dairy cows.

## 2 Results

### 2.1 Sensor Manufacturing

**Figure 1:**
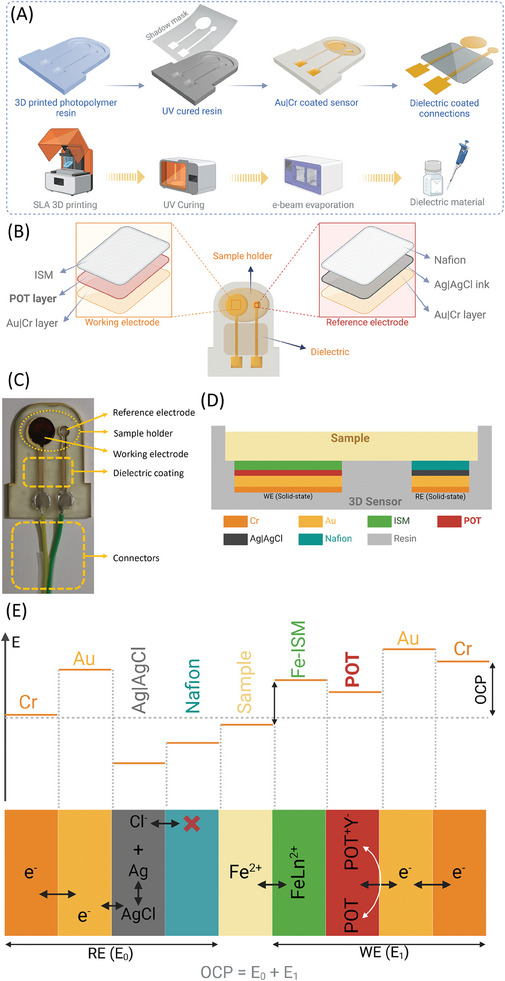
An all‐solid‐state 3D printed ion sensor. A) The sensor manufacturing process involves 3D printing with photopolymer resin, UV curing of the printed sensor, gold and chromium deposition on the uncovered areas using a shadow mask, and applying a dielectric coating to the sensor's wiring. B) The computer‐assisted design (CAD) of the sensor, electrodes, and sample holder, including the WE and RE, involves stacking multiple layers. C) A photograph of a 3D‐printed calcium sensor displaying the WE, RE, dielectric‐coated wiring, and connectors. D) Schematic showing the cross‐sectional view of an iron (Fe) sensor. E) OCP and membrane potential developed at each interface of the 3D‐printed Fe sensor. The overall OCP of the sensor (E) equals the potential at RE (E_0_) plus the potential at WE (E_1_).

This 3D‐printed sensor was manufactured using a stereolithography (SLA) printer, outlined in the Experimental Section. **Figure** **1** illustrates the manufacturing process, sensor structure, and sensing principles.^[^ ^29b^ ^]^ In Figure 1A, the manufacturing process for the sensor base starts with 3D printing the photopolymer resin using an SLA printer. This is followed by curing the resin with ultraviolet (UV) light at a wavelength of 405 nm at 80 °C for 2 h, forming a microscale periodic polymer pattern with a lateral layer width of ≈80 µm and surface wrinkles. We note that the high temp resin used in this study offers a heat deflection temperature (HDT) of 238 °C at 0.45 MPa, according to the manufacturer. This chosen resin facilitates post‐processing techniques like e‐beam evaporation and heat treatment for RE fabrication. The resulting periodic patterns and wrinkles on the sensor's surface create micro‐geometries that enhance the surface area. To investigate the sensor performance, we demonstrated two electrode geometries: one 3D and one 2D. We then conducted cyclic voltammetry (CV) and differential pulse voltammetry (DPV) analyses. In CV measurement (Figure S1A, Supporting Information), the 3D electrode (made of a gold layer) provided a higher current compared to the 2D electrode. A similar observation was noted in the DPV measurements (Figure S1B, Supporting Information), where the current for the 3D electrode was also higher than that for the 2D electrode. Additional details can be found in Figure S1 (Supporting Information).

We have performed surface profilometry on the electrode surface to investigate the periodic microstructures. The resulting profile, shown in Figure S8 (Supporting Information), illustrates the periodic pattern peaks. The average peak height is 2.49 µm, with a standard deviation (SD) of 2.31 µm in printing the height. The variation in micropattern height is due to the profilometer measuring along a 2D line, where any random or wrinkled patterns can significantly affect the height. Next, the sensor was covered with a shadow mask patterned with electrode geometry, and electron‐beam evaporation was used to deposit a 10 nm chromium (Cr) layer as an adhesion material, followed by a 100 nm gold (Au) layer as the electrode.

The connections and wiring were coated with a thin layer of dielectric material to prevent access to these areas, ensuring that modifications were restricted to the circular electrodes only. During the electrode modification steps, such as drop‐casting of POT and ISM, capillary forces caused the liquid solutions to be drawn into the wiring and connection areas. The dielectric coating blocked these regions, limiting the electrode modification to the circular electrode area and improving sensor‐to‐sensor reproducibility. In Figure 1C, the sensor base is designed to have two solid‐state electrodes: the 8 mm diameter working electrode (WE) and the 3 mm diameter reference electrode (RE), each 1 mm deep. The sample holder, 0.3 mm deep, accommodates ≈200 µL of testing sample. The SLA printer enabled the simultaneous production of 20 sensors within 2 h at room temperature. Both electrodes, WE and RE consist of stacked multiple layers, as depicted in Figure 1B,D. A calcium sensor (Ca‐sensor) fabricated is shown in Figure 1C. It depicts the WE, RE, dielectric‐coated wiring, and the connectors. The wires can be connected to the sensor connectors using wire soldering or conductive silver paint. For the RE, a thick layer (5 µm) of silver|silver chloride (Ag|AgCl) was applied over the Au layer, followed by a protonated Nafion layer. The Nafion layer effectively prevents chloride ions from leaching out of the Ag|AgCl layer.^[^ ^40^ ^]^ Figure 1E illustrates the interfacial potentials of a potentiometric Fe‐sensor. The total sensor potential, known as the OCP, denoted as E, combines the constant potential of the RE (E_0_) with the concentration‐dependent potential of the WE (E_1_). The specific ionophore within each ISM generates a membrane potential (E_1_) when exposed to target ions, facilitated by an ion exchange mechanism.^[^ ^14^, ^40^ ^]^ These sensors measure the OCP difference generated between an ion‐selective electrode‐based WE and an RE due to the selective interaction of analyte ions with the ISM.^[^ ^13^, ^15^ ^]^ The voltage at the RE remains constant and is independent of the concentration of the target analyte ions.^[^ ^41^ ^]^ According to the Nernst equation,^[^ ^42^ ^]^ E_1_ at the WE of the sensor directly correlates with the logarithmic activity of the target ions.

### 2.2 Sensor Geometry with Morphology

**Figure 2:**
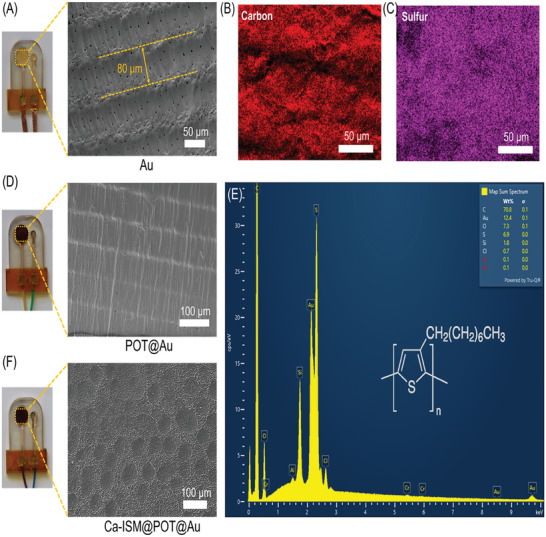
Geometries of the printed sensors. A) Photograph of 3D‐printed sensors after Au coating (top view) and the SEM image revealing randomly oriented small holes that aid in adhering the POT layer. The sensors feature a periodic pattern with each lateral layer width measuring 80 µm. B,C) Carbon and sulfur elemental mapping of the POT‐coated Au surface, respectively. SEM image showing the 3D Au layer with POT layer on printed Ca‐sensor. D) Photograph of the sensor with POT coating on Au‐coated 3D electrodes. The thin POT coating maintained the wrinkled structure of the gold surface E) Energy‐dispersive X‐ray (EDX) spectrum of the POT‐coated Au layer, confirming the presence of various elements post‐POT coating. F) Photograph of a calcium sensor composed of a Ca‐ISM on a POT layer, along with SEM images of the ISM surface. The membrane develops micropores as the cocktail solvent evaporates.

The morphology of sensors along with their elements is investigated using a scanning electron microscope (SEM) and energy‐dispersive X‐ray spectroscopy (EDX). The SEM images reveal the wrinkled gold surfaces on the periodic polymer patterns (**Figure** **2**A), alongside small, nonpatterned microscopic holes within the gold layer formed during the curing process of the 3D‐printed polymer, which was not filled due to the 100 nm thickness of the gold deposition. Separately, a POT solution at a concentration of 1 mg mL^−1^ (see in the Experimental Section) was prepared and applied to the gold layer. The thin POT coating maintained the wrinkled structure of the gold surface (Figure 2D), as observed in SEM images where all holes in the gold film were filled with POT polymer. We note that the wrinkled patterns of the POT layer enhance the sensor's sensitivity and improve the signal‐to‐noise ratio.^[^ ^43^ ^]^ This is because the POT functions as the ion‐to‐electrode transducing interface in this sensor geometry,^[^ ^44^ ^]^ resulting in a higher signal generation. Without the POT layer, the sensor's sensitivity decreases, as shown in Figure S3B (Supporting Information).

The EDX spectrum of the deposited Au layer confirms the presence of carbon (C) and sulfur (S) following POT coating (Figure 2E). The molecular structure of POT is composed of these two elements (see inset of Figure 2E), and the estimated weight percentages of carbon (70.8%) and sulfur (6.9%) in the EDX spectrum. Furthermore, the EDX spectrum reveals substantial amounts of Au within the POT layer (%12.4), indicating a thin POT layer coating on the electrode surface. The elemental mapping analysis of the gold electrode coated with POT in Figure 2B,C demonstrates a uniform coating of the POT layer. The capillary force of tetrahydrofuran, used as the solvent for the POT solution, contributes to the uniform distribution of POT on the gold electrode.^[^ ^45^ ^]^ In this study, four different ion‐selective membrane (ISM) cocktail solutions were separately prepared for Fe^2+^ (Fe‐Sensor), NO_3_^–^ (N‐Sensor), Ca^2+^ (Ca‐Sensor), and PO_4_^3–^ (P‐Sensor). The primary component of all these membranes is polyvinyl chloride (PVC).^[^ ^46^ ^]^ This polymer matrix encases the ionophore. Figure 2F shows the electrode coated with the membrane (Ca‐ISM). The microporous membrane forms as the cocktail solvent (THF) evaporates, which further enhances the sensor's sensitivity by expanding the membrane's surface area (Figure 2F). Detailed preparation methods for these ISMs are provided in Experimental Section. Each sensor's WE was prepared by applying 30 µL of the respective cocktail solution onto the surface of the POT film. Detailed principles of operation for all sensors (Fe‐Sensor, Ca‐Sensor, N‐Sensor, and P‐Sensor) are also outlined in Experimental Section.

### 2.3 Sensing of Multiple Ions Using 3D‐Printed Sensors

**Figure 3:**
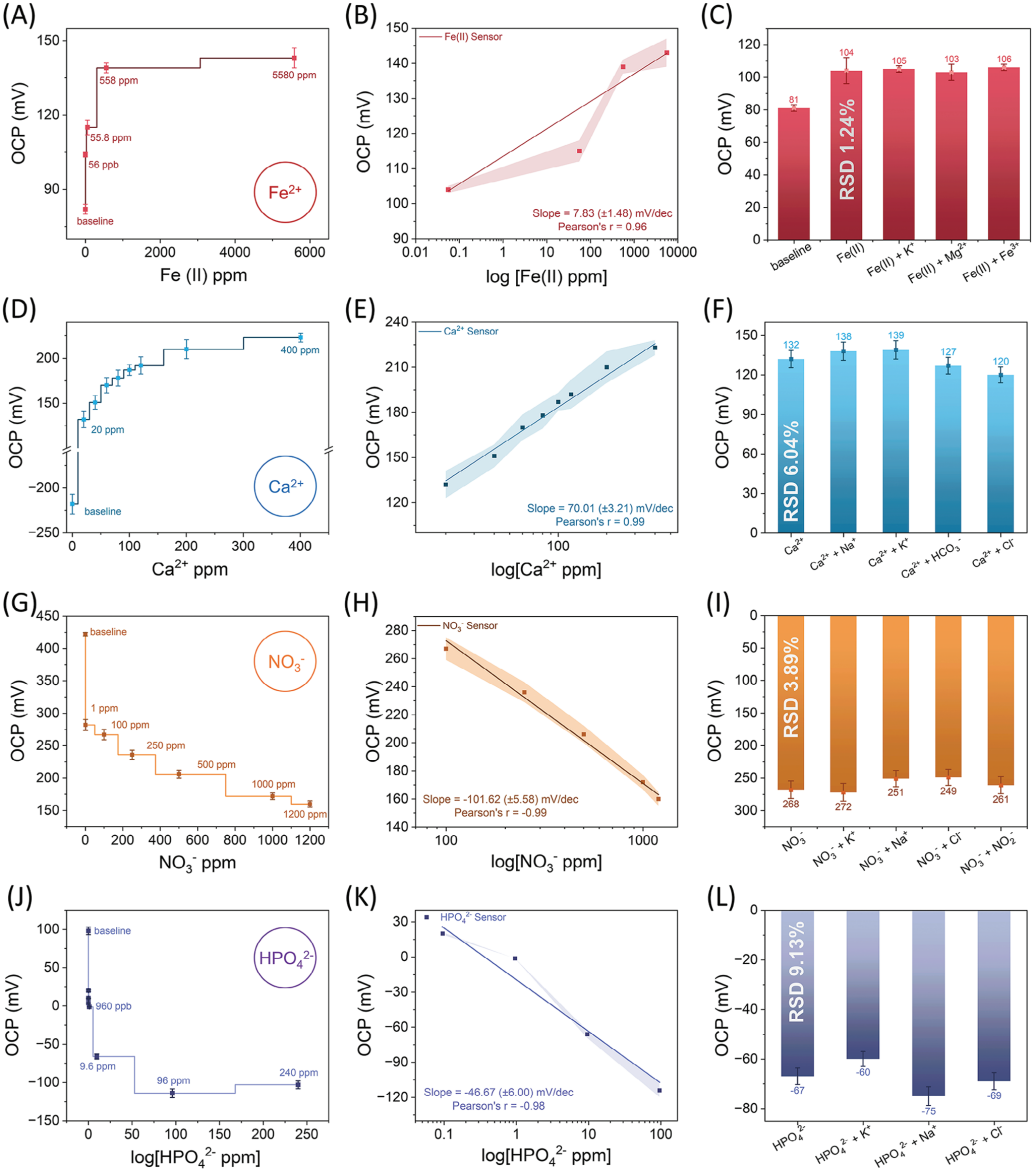
Sensor performance for detecting multiple ions. A) For Fe‐Sensor, dose‐dependent solutions (56 ppb–5580 ppm) of Fe^2+^ were tested. B) Calibration plots of F‐Sensor (Pearson's r = 0.96). C) The selectivity study of the F‐sensor in the presence of Fe^3+^ (40 ppm), K^+^ (39 ppm), and Mg^2+^ (23.4 ppm). These ions were combined with a fixed concentration of iron (II) ion (56 ppb) in a 1:1 ratio to evaluate the sensor's discrimination capability against interferents. The RSD was determined to be 1.24%. D) For the Ca‐Sensor, dose‐dependent solutions (20–400 ppm) of Ca^2+^ were tested. E) Calibration plots showed Pearson's r = 0.99. F) The selectivity study involved testing the sensor's discrimination capability against potassium ions (200 ppm), chloride ions (200 ppm), bicarbonate ions (200 ppm), and sodium ions (200 ppm), combined with a fixed concentration of calcium ions (20 ppm) in a 1:1 ratio. The RSD was determined to be 6.04%. G) For the N‐sensor, dose‐dependent solutions (1–1200 ppm) of NO_3_^−^ were tested. H) Calibration plot: Pearson's r = −0.99. I) Selectivity study involved testing against potassium ions (100 ppm), chloride ions (100 ppm), nitrite ions (100 ppm), and sodium ions (100 ppm), combined with a fixed nitrate ion concentration (50 ppm) in a 1:1 ratio to evaluate interference discrimination capability. The RSD was determined to be 3.89%. J) For the P‐sensor, dose‐dependent solutions (960 ppb–240 ppm) of HPO_4_^2−^ were tested. K) Calibration plot: Pearson's r = −0.98. L) Selectivity study included testing against potassium ions (50 ppm), chloride ions (50 ppm), and sodium ions (50 ppm), combined with a fixed hydrogen phosphate ion concentration (94 ppm) in a 1:1 ratio to evaluate interference discrimination capability. The RSD was determined to be 9.13%. The precision of each sensor in sensing, calibration, and selectivity studies was assessed by calculating the standard deviation (SD) based on three independent measurements (*n* = 3). The results are represented visually using error bars and shaded error regions.

**Figure 4:**
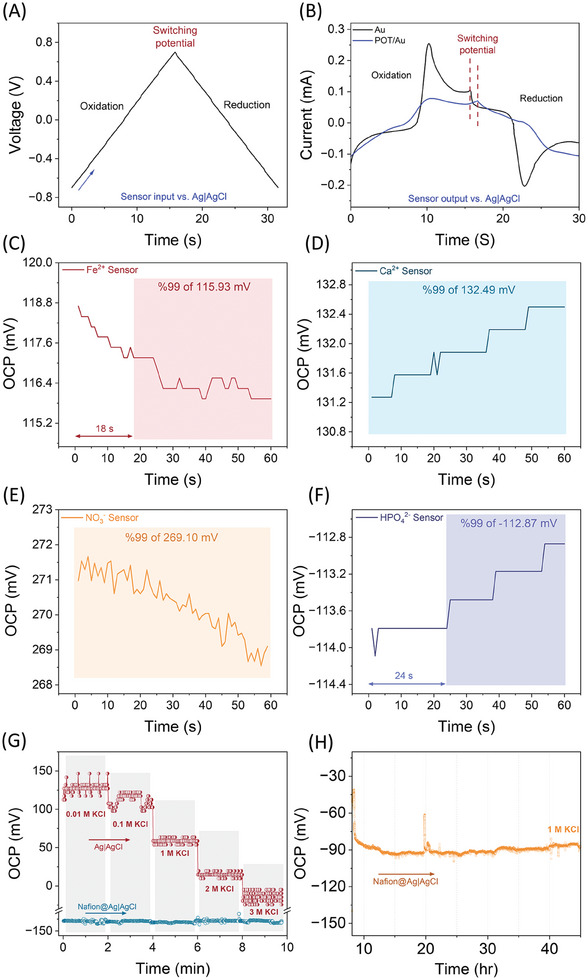
Electrochemical characterizations, detection time, and sensor stability. A) Cyclic voltammetry (CV) tests were conducted by applying a voltage waveform between the WE and RE for 30 s, ranging from −0.7 to 0.7 V. B) The resulting current waveforms from sensors with Au and POT layers were recorded at a scan rate of 50 mV s^−1^. These CV tests were performed in PBS (pH 7.4) containing a mediator, 5 mm Fe(CN)_6_^3−/4−^, at an equimolar concentration of 5 mm. C) The OCP of the Fe‐sensor in a standard solution containing 55.8 ppm of Fe^2^⁺ was used to calculate the detection time. The sensor reached 99% of its response within 18 s. D) The OCP of the Ca‐sensor in a standard solution containing 20 ppm of Ca^2^⁺ was used to calculate the detection time. The sensor reached 99% of its response immediately at the beginning of the experiment. E) The OCP of the N‐sensor in a standard solution containing 100 ppm of NO_3_^−^ was used to calculate the detection time. The sensor reached 99% of its response immediately at the beginning of the experiment. F) The OCP of the P‐sensor in a standard solution containing 96 ppm of HPO_4_^2^^−^ was used to calculate the detection time. The sensor reached 99% of its response within 24 s. G) The fabricated RE of Ag|AgCl with and without Nafion was chosen to investigate the Cl^–^ activity with various concentrations of 0.01, 0.1, 1, 2, and 3 m KCl. A commercial RE was taken to measure the OCP of fabricated RE. The protonated Nafion on the Ag|AgCl layer does not allow to leach Cl^–^ and provides more stability. H) Long‐term measurement (45 h) of fabricated RE (Nafion@Ag|AgCl) in the presence of 1 m concentration of KCl.

The sensing performance, selectivity capabilities, and calibration equation for each sensor targeting specific ions have been evaluated using OCP measurements between the WE and the RE. Figure 3 demonstrates the potentiometric sensing results, calibration graphs, and the selectivity performance for multiple ions, including Fe^2+^, NO_3_^–^, Ca^2+^, and HPO_4_^2–^. The Fe‐sensor monitored the OCP for over 60 s between electrodes, with Fe^2+^ titrate concentrations ranging from 56 ppb (low) to 5580 ppm (high). In DI water, the Fe‐sensor baseline was obtained at 82 mV without any target Fe^2+^. Upon the introduction of 56 ppb Fe^2+^, the sensor signal increased to 104 mV, indicating an interaction between the Fe ions and the Fe‐ISM, leading to the observed potential development associated with Fe^2+^ ions. As Fe^2+^ concentrations further increased from 55.8 to 5580 ppm, the sensor's OCP demonstrated a direct proportional increase. The sensor was rinsed with DI water before being exposed to a new concentration. Calibration of the Fe‐sensor involved plotting OCP values (Figure 3B) against Fe^2+^ concentrations, employing logarithmic scales. This analysis effectively characterized the relationship between the sensor's OCP and varying Fe^2+^ concentrations. For most target concentrations, the Fe‐Sensor reaches saturation within 18 s, indicating rapid detection of Fe^2+^ (**Figures** **3**A and **4**C). Each of the four sensors has been examined in terms of response time and will be discussed in the following section. The shaded error region (SER), representing the standard deviation of three independent measurements (*n* = 3), was calculated to assess the precision of the sensors.

Next, we evaluate the performance of the N‐Sensor for the Ca‐Sensor for calcium ion, detection the nitrate ion detection, and the P‐Sensor for the detection of hydrogen phosphate ion. Each sensor utilized a POT layer as a transduction material along with a specific ISM layer tailored to its target ion. The calibration of these sensors was carried out using dose‐dependent titrate solutions to establish their quantitative analytical capabilities. Dose‐dependent solutions of nitrate, calcium, and hydrogen phosphate ions were prepared by diluting stock solutions in deionized water to concentrations ranging from 1 to 1200 ppm for nitrate, 20 to 400 ppm for calcium, and 94 ppb to 94.7 ppm for hydrogen phosphate, respectively. The N‐Sensor, Ca‐Sensor, and P‐Sensor were employed to measure the OCP responses in the presence of these solutions. Each measurement was independently repeated three times (*n* = 3) to ensure reproducibility and evaluate the error bars. Sensing measurements were conducted using the Ca‐Sensor in DI water, spanning concentrations of Ca^2+^ ions from 20 to 400 ppm. The baseline signal of the Ca‐Sensor was found at −217 mV, and upon exposure to 20 ppm of calcium concentration, this value increased to 133 mV. Across the range of up to 400 ppm of calcium concentration, the Ca‐Sensor consistently exhibited a proportional response to the Ca levels (Figure 3D). Like the Fe‐Sensor, the Ca‐Sensor incorporates a specific Ca‐ISM with an ionophore designed to selectively capture Ca^2+^ ions from the analyte solution. Calibration plots, both with and without logarithmic transformation, demonstrated high linearity, yielding a Pearson's r value of 0.99 (Figure 3E). The analytical sensitivity of the Ca‐Sensor was found to be below 20 ppm concentration.

The OCP responses of the N‐Sensor in the presence of various nitrate ion concentrations exhibited a clear dose‐dependent relationship (Figure 3G). The baseline signal of the N‐Sensor was initially found at −421 mV in DI water. Upon testing with a nitrate ion concentration of 1 ppm, the OCP decreased to −281 mV, indicating a detectable response to the presence of nitrate ions. (Figure 3G). Further, as we increased the nitrate concentration from 1 to 1200 ppm, the N‐sensor OCP decreased from 281 to 160 mV. This change is attributed to the nitrate ionophore present in the nitrate‐ISM on the sensor surface, which selectively binds nitrate ions from the samples, thereby generating a corresponding potential response.^[^ ^47^ ^]^ Calibration plots of N‐Sensor with logarithmic transformation showed high linearity (Pearson's r = −0.99), indicating robust performance across the tested concentration range (Figure 3H). The analytical sensitivity of the N‐sensor is 1 ppm concentration. In addition to the sensors designed for iron, calcium, and nitrate, a hydrogen phosphate ion‐selective membrane (P‐ISM) is integrated into the POT surface of the P‐Sensor for phosphate detection. The baseline signal of the P‐Sensor exhibited an initial OCP response of 112 mV. Upon introduction of a phosphate concentration of 94 ppb, the OCP of the P‐Sensor decreased to 21 mV (Figure 3J). Subsequently, testing with a higher concentration of 96 ppm phosphate resulted in a corresponding OCP variation to −111 mV. The P‐Sensor demonstrated robust responsiveness to phosphate ions, as evidenced by a calibration plot with a high coefficient of determination (Pearson's r = −0.96) (Figure 3K). The shaded error region, representing the standard deviation from three independent measurements, was included to illustrate the precision of the sensors for N, Ca, and P ions. The analytical sensitivity of the P‐Sensor was determined to be 94 ppb concentration. We note that the physiological concentration of HPO₄^2^⁻ in wastewater or river water is typically less than 5 ppm.^[^ ^48^ ^]^ The developed P‐sensor can easily detect this concentration range, making it suitable for assessing water quality in river or wastewater treatment plants.

The selectivity of each sensor was evaluated in the presence of the most likely interfering ions that could affect the sensor's performance in real‐world environments. We selected ion concentrations that were equal to or higher than those found in various real environmental, clinical, or biological samples, such as milk, lake water, or industrial wastewater. Figure 3C,F,I,L presents the results of selectivity studies conducted for the Fe‐sensor, Ca‐Sensor, N‐sensor, and P‐Sensor, each evaluated under controlled conditions to assess their specificity. The Fe‐sensor demonstrated exceptional selectivity toward nonspecific target ions, as evidenced in Figure 3C. To evaluate its selectivity, interference studies were conducted using iron (III) (40 ppm), potassium (39 ppm), and magnesium (23.4 ppm) as common interfering ions, deliberately chosen at elevated concentrations. These interferents were mixed in a 1:1 ratio with a fixed Fe^2+^ concentration of 56 ppb, which is chosen to be ≈1000 times lower in concentration compared to interfering ions The chosen concentrations are selected to encompass the ion levels found in river water, taking into account their practical applications. The typical ionic concentrations in ppm for Fe^2^⁺, Mg^2^⁺, and K⁺ are 0.01–1.0, 1–100, and 0.5–10 in river water, respectively, and less than 0.3, 30, and 10 in high‐quality drinking water, respectively. When dissolved oxygen levels exceed 1–2 ppm, iron exists predominantly as Fe^3+^, whereas at lower oxygen concentrations, it is found as Fe^2+^. Fe^2+^ is highly soluble, while Fe^3+^ is not, and the equilibrium between these two forms depends on the oxygen content in the water. The resulting relative standard deviation (RSD) of ≈1.24% highlights the sensor's robust capability to accurately detect Fe^2+^ in the presence of interferents. This high selectivity is primarily attributed to the incorporation of a specific Fe^2+^ ionophore within the Fe‐ISM. This specialized ionophore facilitates the preferential exchange of Fe^2+^ ions while effectively inhibiting interactions with nonspecific targets. As a result, the Fe‐sensor ensures precise and reliable detection of Fe^2+^, underscoring its suitability for sensitive analytical applications. Figure 3F depicts the Ca‐Sensor's response to a target concentration of 20 ppm calcium, where potassium, sodium, and chloride ions were present as nontarget interferents at 200 ppm each, in a 1:1 ratio. In cow's milk, the concentration of calcium ions is roughly equal to that of other ions. In contrast, in bovine serum, calcium ion concentration is about one‐tenth that of sodium, potassium, and chloride ions. Therefore, we have selected the concentration of interfering ions to be ten times higher than that of calcium. The relative standard deviation (RSD) of the Ca‐Sensor in the presence of these interferents was found to be 6.04%, highlighting its high selectivity. In Figure 3I, the N‐Sensor was tested with a target concentration of 100 ppm nitrate, also mixed with potassium, sodium, chloride, and nitrite ions at 50 ppm each. In agriculture runoff, typical ionic concentrations in ppm are ≈5–100 for nitrate ions, 1–50 for sodium, potassium, and chloride ions, and 1–10 ppm for nitrite ions that are well covered by our proposed N‐sensor. The RSD for the N‐Sensor under these conditions was determined to be 3.89%, indicating its excellent selectivity. Similarly, Figure 6L demonstrates the performance of the P‐Sensor with a target concentration of 94 ppm phosphate, under the same mixing conditions of potassium, sodium, and chloride ions at 50 ppm each. The P‐Sensor exhibited an RSD of 9.13%, confirming its good selectivity.

We also investigated the impact of pH on ion sensing. As shown in Figures S7A,B (Supporting Information), pH has a minimal effect on iron and calcium detection. Nevertheless, it is important to calibrate the sensor using solutions that closely match the intended application. Phosphate, being a polyprotic acid, has many forms whose concentrations vary with pH, corresponding to the phosphate's fractional composition^[^ ^49^ ^]^ (Figure S7C,D, Supporting Information). P‐Sensor demonstrates that the sensor's response follows a sigmoidal curve shape with pH changes, reflecting variations in HPO_4_^2−^, H_2_PO_4_^−^, and PO_4_^3−^ concentrations. The pH range used in our experiments is highlighted in the fractional composition graph (Figure S7D, Supporting Information). More details are provided in the Supporting Information.

Based on the detailed evaluation of Fe, Ca, N, and P sensors based on the wrinkled POT layer for detecting respective ions, it is evident that each sensor demonstrates robust performance in terms of sensing accuracy, calibration reliability, and selectivity against interfering ions. The sensors exhibit high sensitivity across a range of concentrations, as indicated by their calibration plots and Pearson's correlation coefficients. Furthermore, their ability to maintain low RSDs in the presence of significant interferents indicates their suitability for practical applications. The observed high selectivity can be attributed to the specific ISM and the hydrophobic POT layer that effectively repels other ions from the sensor interface. The contact angle of 72° displayed in Figure S3A (Supporting Information) illustrates the hydrophobic properties of the POT. The hydrophobic property of polymer POT is critical in improving sensor selectivity by repelling interfering ions, stabilizing the sensor interface, selectively permitting target ions, and reducing background noise.^[^ ^50^ ^]^ Specifically, the hydrophobic nature of solid‐contact POT creates a barrier that prevents nontarget ions from samples from approaching or accumulating at the interface of POT/ISM or POT/Au.^[^ ^50^, ^51^ ^]^ We noted that such characteristics are crucial for the sensors' reliability in various environmental, clinical, agricultural, and livestock applications where precise ion detection is essential.

### 2.4 Electrochemical Studies

To investigate the specific redox properties imparted by a POT layer in four different sensors, we conducted cyclic voltammetry (CV) tests lasting 30 s. This test involves applying a potential waveform (Figure 4A) between the WE and RE, sweeping linearly from −0.7 to +0.7 V at a constant scan rate of 50 mV s^−1^, with a subsequent reverse sweep to the initial potential. The resulting CV output waveform (Figure 4B) depicts the current plotted against applied potential. During the potential sweep, electrochemical reactions occur at the surfaces of both POT‐modified and bare Au electrodes, reflecting electro‐catalytic activity toward oxidation and reduction processes of mediator at the electrode interface. Notably, the sensor with a bare Au electrode exhibited higher oxidation or reduction peaks owing to the excellent conductivity of the gold metallic layer. Precious metals like platinum and gold exhibit excellent electrocatalytic activity toward the ferro/ferricyanide (Fe(CN)_6_^3−/4−^) redox reaction due to their favorable electronic properties. These metals provide efficient electron transfer kinetics and stable surface adsorption sites, which facilitate the redox processes. Additionally, their resistance to corrosion and surface oxidation ensures long‐term catalytic performance and reliability. Introducing a POT layer onto the gold surface suppressed the electrochemical response approximately threefold (Figure 4B). Despite POT's favorable redox properties, its negatively charged layer repels ferro/ferricyanide molecules, resulting in reduced current output. Despite the decrease in electrochemical current observed with the POT layer, these redox properties are valuable for sensors because the electrochemical reaction in potentiometric sensors is minimal, it generates almost no current during the measurement process. This study indicates the capability of POT as a modifier of electrode characteristics in potentiometric sensor applications, providing valuable insights into enhancing sensor sensitivity and selectivity through surface modifications with ion‐selective membranes, as illustrated in Figure 1D.

We conducted an Electrochemical Impedance Spectroscopy (EIS) experiment on the sensor with and without the POT layer on the gold electrode. The resulting Nyquist plots are shown in Figure S2 (Supporting Information). From these plots, we calculated the charge transfer resistance (*R*_ct_). Specifically, the Rct value for the bare gold electrode was found to be 233.6 Ω, whereas the POT‐coated gold electrode exhibited an *R*_ct_ of 4808.3 Ω. This increase in *R*_ct_ is attributed to the POT layer, which acts as a resistive material, impeding the transfer of electrons from the electrolyte.

### 2.5 Detection Time, Long‐term Stability, and Repeatability

To measure the response time of four sensors, we designed and conducted an experiment for each one. During the experiment, each sensor measured a standard solution of the target ion for 60 s without any incubation. We took the OCP signal at the 60th s as the sensor's response signal and calculated 99% of this signal to determine the response time. The moment each sensor first reached this 99% signal, we recorded the time as the sensor's response time. In this context, the Fe‐sensor was tested using a 55.8 ppm Fe^2+^ standard solution, with the resulting OCP response shown in Figure 4C. The OCP signal of the Fe‐sensor at 60 s into the experiment measured 115.93 mV, and the sensor reached 99% of this signal after 18 s. Thus, the response time of the Fe‐sensor is 18 s. The same experiment was conducted for the Ca‐sensor, N‐sensor, and P‐sensor using standard solutions of 20 ppm Ca^2+^, 100 ppm NO_3_^−^, and 96 ppm HPO_4_^2−^, respectively. These sensors achieved 99% of their final signal immediately at the beginning of the experiment for Ca‐sensor and N‐sensor and 24 s from the start of the experiment for the P‐sensor. This demonstrates the rapid response nature of all four sensors, attributed to the ISM that quickly reaches equilibrium with the targeted ions. The thin ISM layer also reduces the path length required for the ion to reach the ionophore and reduces the time needed for the sensor to reach equilibrium, enhancing its response speed. Additionally, the flexible polymer chains in POT quickly adapt to changes in the ionic environment in the adjacent ISM layer, allowing the sensor to achieve equilibrium faster after exposure to the target ions. Furthermore, the oxidation of POT is a surface‐confined process, which means that the redox reactions are restricted to the surface layer of the POT, thereby accelerating the overall response time.

Figure 4G presents stability and long‐term measurement data for the Nafion on Ag|AgCl (Nafion@Ag|AgCl) reference electrode (RE), focusing on its performance under varying chloride ion concentrations. The sensor stability was verified by investing the sensor's response with chloride ion activity as the chloride ion leaching from RE is a major concern. In Figure 4G, the fabricated RE (Ag|AgCl) with and without Nafion coating was utilized to assess chloride ion activity. The chloride ion concentrations ranging from 0.01 to 3 m were prepared for this study. The Ag|AgCl electrode with protonated Nafion coating was specifically chosen to mitigate chloride ion leaching, thereby enhancing stability in the sensor's response. Nafion coating effectively maintains chloride ion activity and minimizes potential interference, crucial for accurate electrochemical measurements. The fabricated RE exhibited a stable OCP when coated with Nafion across a range of chloride ion concentrations from 0.01 to 3 m. In contrast, the RE sensor without Nafion showed varying OCP values in response to different chloride ion concentrations. Figure 4H illustrates the results of long‐term continuous measurements over 45 h using the Nafion‐coated RE immersed in a 1 m chloride ion solution. This extended‐duration study provides insights into the electrode's performance stability under continuous exposure to a high chloride ion concentration. Initially, within ≈15 h of sensor preconditioning, the OCP changed from −40.5 to −93.4 mV. Subsequently, over the next 30 h, the OCP remained nearly unchanged at ≈ −93 mV, indicating a stabilization rate of ≈±1 to 2 mV per hour. The long‐term measurement data in Figure 4H confirm the electrode's robustness in maintaining stable OCP readings over an extended period in high chloride ion environments, highlighting its suitability for applications requiring continuous monitoring.

Additionally, we have conducted repeatability tests on all four sensors by measuring five different standard solutions of the target analyte and calculating the relative standard deviations (RSDs) of the results. As shown in Figure S4 (Supporting Information), the Fe sensor exhibited a low RSD of 3.14% for repeatability tests using a 1 ppm Fe^2^⁺ standard solution. The Ca^2^⁺, N, and P sensors demonstrated RSDs of 1.16%, 1.39%, and 0.75% for the measurement of 200 ppm Ca^2^⁺, 500 ppm NO₃⁻, and 100 ppm HPO₄^2^⁻, respectively. These low RSD values indicate that all four sensors can produce repeatable results under the same conditions over multiple trials (five times). This testing verifies the precision and reliability of the sensors by ensuring that their readings remain stable and reproducible across different measurements.

### 2.6 Sensor Integration with Microfluidic Devices in a Milking Parlor

This study integrates microfluidic devices to enable real‐time analysis of milk samples, providing precise control and manipulation of fluid volumes.^[^ ^52^ ^]^ The seamless interfacing with milking machines enhances the efficiency and reliability of milk quality assessment. By incorporating electrochemical sensors, this integrated approach enhances sensitivity, specificity, and reproducibility, enabling the detection of low‐abundance analytes in complex matrices. These advancements support real‐time monitoring for applications in point‐of‐care diagnostics, personalized medicine, and environmental assessments, contributing to significant advancements in dairy production and quality control practices. Furthermore, temperature (*T*) plays a critical role in the potentiometric sensors with ISMs, as governed by the Nernst equation (Equation 1).^[^ ^53^ ^]^ This equation describes the potential difference across the membrane as a function of ion concentration, with temperature being a key factor. As temperature increases, the mobility of ions and the membrane's response typically become more pronounced, affecting the accuracy and precision of the measurements. Proper temperature control and calibration are essential to ensure reliable and reproducible results, as deviations can lead to significant errors in detecting ions. Understanding and compensating for temperature effects are thus fundamental in achieving accurate potentiometric analyses of all of the four proposed sensors. For example, milk samples from a milking machine or a portable milker are warmer than at room temperature, and using a sensor calibrated with standard solutions at room temperature might lead to significant errors in ion detection. This issue also arises in other applications, such as wastewater measurement.

(1) $$E=E^{0}\;-\frac{RT}{nF}\ln a$$

To assess the impact of temperature fluctuations on the accuracy and precision of the sensor, we conducted a validation test using the Ca‐sensor as a model system. The test was performed with three standard solutions, each containing 40 ppm Ca^2+^, and measurements were taken at three different temperatures: 10.0, 25.0, and 40.0 °C. As shown in Figure S5A,B (Supporting Information), temperature notably affects both the accuracy and precision of the measurements by influencing the potential difference between the working and reference electrodes.

**Figure 5:**
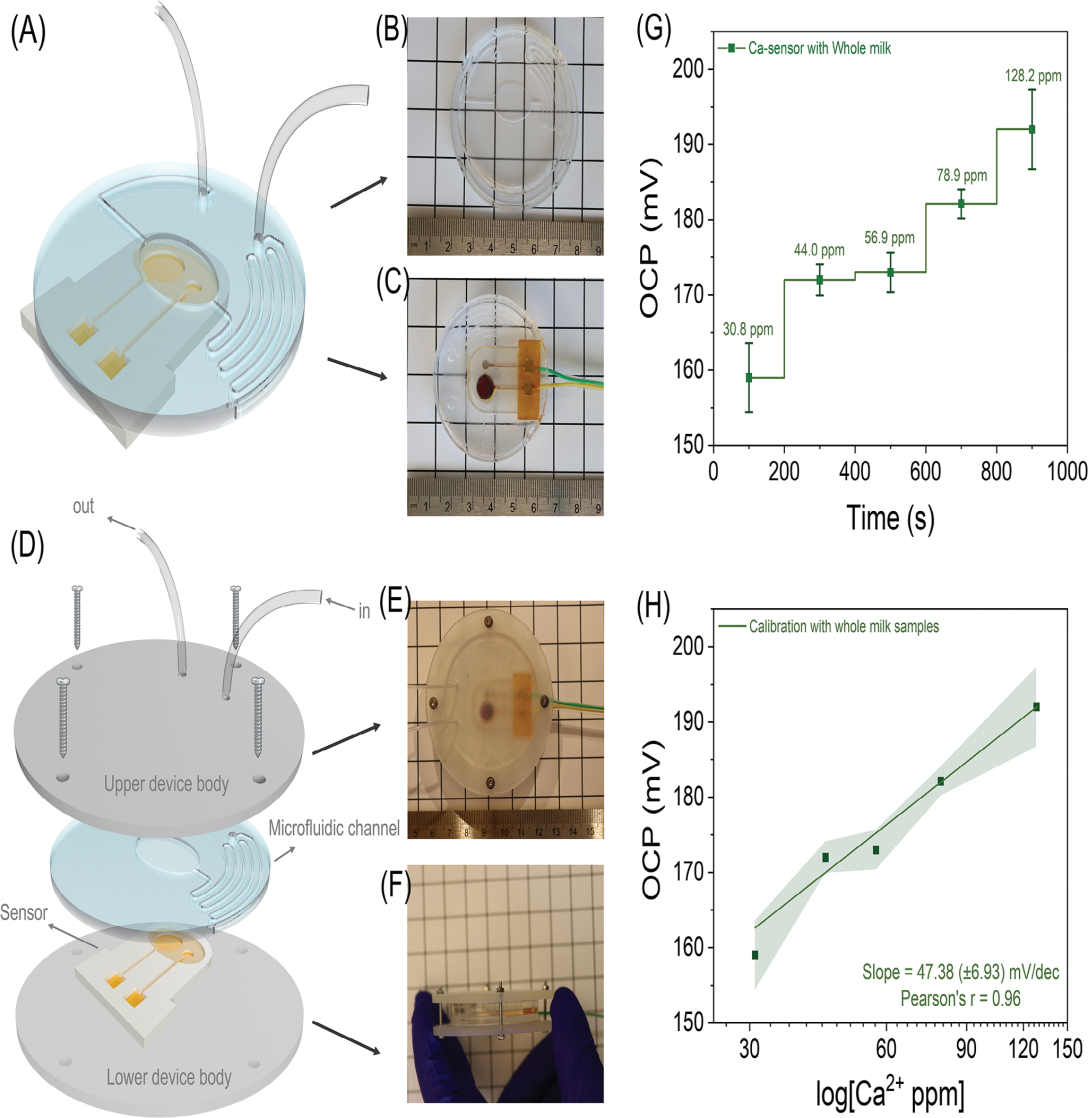
Microfluidic device integration with the 3D‐printed sensor. A) The schematic illustrates a PDMS microfluidic channel positioned above the 3D‐printed Ca‐sensor. The serpentine microchannel has a diameter of 500 µm and a length of ≈171 mm to ensure uniform temperature distribution of fresh milk from the cow's body for all samples. The microchannel extends to the 3D sensor's sample holder, which shares the same oval shape. B) An image of the PDMS microfluidic system fabricated as a negative replica mold. C) An image of the calcium sensor integrated with the PDMS microfluidic system. D) A schematic of the Ca‐sensor device, which features a top and bottom 3D‐printed body made of clear resin, encasing the 3D sensor and the PDMS microfluidic system, and secured with screws. E) A top‐view photo of the device. F) A side view photo of the device. G) The OCP of whole milk spiked with a standard concentration of calcium, measured using a commercial calcium ion meter. H) The calibration graph was obtained from whole milk samples using a 3D‐printed sensor. The Pearson's r was determined to be 0.96. The precision of each sensor in sensing and calibration studies was assessed by calculating the standard deviation (SD) based on three independent measurements (*n* = 3). The results are represented visually using error bars and shaded error regions.

To equalize the temperature of milk samples between lab and farm measurements and reduce sample‐to‐sample temperature and volume fluctuations, we designed a polydimethylsiloxane (PDMS) microfluidic system with a serpentine microchannel of 500 µm diameter and 171 mm length (**Figure** **5**A). This serpentine channel allows the milk sample to cool down while the increased surface‐to‐volume ratio of the microchannel enhances exposure to the cool channel walls. To evaluate the cooling efficiency of the microfluidic system using deionized water, we conducted an experiment (Figure S6, Supporting Information) in which we measured the fluid temperature at both the inlet and outlet. The results presented in Table S1 (Supporting Information) demonstrate the system's effectiveness in lowering the temperature. The design of the long channel facilitates heat dissipation, bringing the sample to room temperature.

Additionally, we ensure a controlled volume of the sample is pumped onto the sensor's surface to minimize sample‐to‐sample fluctuations, improving repeatability. The PDMS channel was fabricated using a 3D‐printed master mold, and the PDMS microfluidic system is shown in Figure 5B. The CAD design of the sensor and microfluidic system are complementary, with the microfluidic system featuring a sample reservoir precisely aligned with the oval sample holder of the sensor. Figure 5C shows the integration of the 3D‐printed sensor with the microfluidic system. For farm deployability and to ensure mechanical stability for unsupervised end‐users, we designed a robust, pocket‐size device that integrates and embeds both the sensor and the PDMS microfluidic system. Figure 5D illustrates this design, which includes 3 mm diameter holes for screws and 1.5 mm diameter holes for the microtube inlet and outlet. The device body was printed using photopolymer resin, with the detailed manufacturing procedure provided in Experimental Section. Figure 5E,F displays the top and side views of the assembled device, showcasing the integrated Ca‐sensor and microfluidic system. The two wires protruding from the device connect to the RE (green) and WE (yellow). This durable sensor is paired with a miniaturized peristaltic micropump, powered by a 3 V battery, capable of pumping 300 µL min^−1^.

We evaluated the Ca‐sensor using whole milk samples to detect calcium in this complex matrix. The samples were spiked with calcium ions, and their calcium levels were measured in ppm using a commercial calcium meter. The calcium levels in the five milk samples were 30.8, 44.0, 56.9, 78.9, and 128.2 ppm. The Ca‐sensor successfully detected the differences in calcium levels, as shown by the OCP in Figure 5G. The calibration graph for these milk samples, displayed in Figure 5H, has a Pearson's r of 0.96, demonstrating the sensor's ability to produce a linear response according to the Nernstian equation, even in the complex matrix of milk.

**Figure 6:**
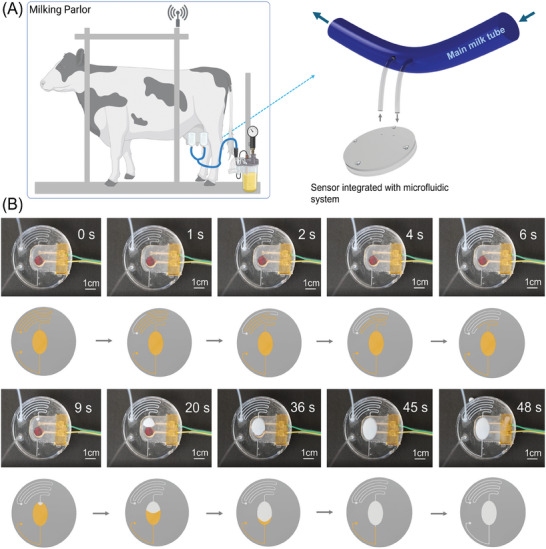
The microfluidic integrated Ca‐sensor was interfaced into a milking parlor. A) An instance of using the sensor device in a milking parlor involves its Ca‐sensor for real‐time monitoring of milk calcium levels. This application serves to enhance milk quality control and detect postpartum hypocalcemia efficiently. B) Real‐time snapshot images show the microfluidic system with a 3D‐printed calcium sensor in action as it pumps real milk samples. Each image is accompanied by a schematic that indicates the volume of milk being pumped (via a peristaltic micropump) into the system. Additionally, an inset in each image displays the elapsed time during the pumping process.

**Figure** **6** shows the microfluidic integrated Ca‐sensor interfaced into a milking parlor. An instance of using the sensor device in a milking parlor involves its Ca‐sensor for real‐time monitoring of milk calcium levels (Figure 6A). This application serves to enhance milk quality control and detect postpartum hypocalcemia efficiently. According to Figure 6B, the milk sample spends ≈10 s in the microchannels before reaching the reservoir region for sensing. The sensing region is also filled with milk samples after 35 s, and the entire process of cooling down and pumping takes enabled by using a peristaltic micropump for less than 50 s. As previously mentioned, the full sensing cycle with the OCP technique takes 60 s, although sensors exhibit faster detection times. Therefore, the complete sensing procedure takes less than 2 min. Moreover, continuous pumping of the sample enables online monitoring. As previously discussed, the N‐sensor and Ca‐sensor can achieve 99% of their signal immediately, while the Fe‐sensor and P‐sensor generate their responses 18 and 24 s, respectively, after coming into full contact with the sample. This rapid reaction, coupled with the capabilities of the microfluidic system, allows our sensing device to be suitable for continuous and real‐time monitoring across diverse applications.

## 3 Discussion

POT's oxidation process is surface‐confined and rapid, ensuring the membrane‐sample interface remains near thermodynamic equilibrium across a broad concentration range. This equilibrium allows for simplified theoretical predictions of sensor behavior without extensive diffusion coefficient considerations, enhancing sensor robustness and design simplicity. POT‐based membranes also achieve low detection limits, similar to traditional stripping voltammetry, making them effective at low sample concentrations. These features make POT‐based sensors highly effective for precise and reliable ion detection, with applications in clinical analysis and multianalyte detection in complex samples like human serum, milk and water samples.

Additionally, our sensors show a very fast response which is due to both the POT layer and the thin ISM layer atop it. The thin membrane, often in the micrometer to sub‐micrometer range, significantly reduces the diffusion distance for ions. This allows for rapid ion transfer across the membrane‐sample interface. Additionally, the oxidation of POT is a surface‐confined process, meaning that the redox reactions are limited to the surface layer of the POT, further speeding up the overall response time. This makes the sensor favorable for applications in continuous measurements and online monitoring.

Extrusion printing via stereolithography is used for lateral layer‐by‐layer deposition of high‐temperature resin, creating periodic wrinkled structures on the electrode surface during curing. This 3D printing method allows for the fabrication of sensor bases, the proposed device, and the master mold for microfluidic system materials, enabling complex geometries, controlled structures, and a variety of material combinations.^[^ ^28a^ ^]^ Importantly, it circumvents the costly clean room microfabrication processes (such as photolithography or e‐beam lithography) typically used for sensor prototyping. Unlike other 3D printing methods, including direct nanoparticle printing,^[^ ^32^, ^54^ ^]^ this approach is cost‐effective and maintains excellent sensor reproducibility while offering superior sensing properties. The wrinkled surface topology in our printed sensors provides a high surface area conducive to incorporating conducting polymers like POT, thereby enhancing sensing performance including limit‐of‐detection and response time compared to traditional 2D sensing geometries. 3D electrodes enhance analyte diffusion compared to 2D electrodes due to their increased surface area, which provides more active sites for reactions, and their structure, which facilitates better mass transport. The periodic wrinkled structures of 3D electrodes allow ions to diffuse more efficiently, while the geometry promotes convective flows that improve transport to the electrode surface, reducing diffusion layer thickness. Additionally, the shorter diffusion paths in 3D electrodes speed up the analyte's access to reactive sites, and the full utilization of the electrode material increases overall performance, making diffusion faster and more efficient than in 2D electrodes.

The all‐solid‐state 3D‐printed sensors based on a solid‐contact POT layer underwent comprehensive characterization, including electrochemical testing, to evaluate their multi‐ion sensing and selectivity performances. Figure 3 demonstrates potentiometric sensing results for multi‐ions such as Fe^2+^, NO_3_^–^, Ca^2+^, and HPO_4_^2–^ wherein the solid‐contact POT layer acted as a universal ion‐to‐electron transducing layer for all sensors. The Fe‐sensor (Figure 3b) exhibited a clear relationship between OCP and Fe^2+^ concentrations, reaching saturation within 18 s for most concentrations, indicating the sensor detection time is less than 20 s without any incubation (Figure 4C). Interference studies (Figure 3C) demonstrated the Fe‐sensor's exceptional selectivity, showing an RSD of ≈5.78% when tested against common interferents. Similarly, the N‐Sensor, Ca‐Sensor, and P‐Sensor displayed dose‐dependent responses to nitrate, calcium, and hydrogen phosphate ions, respectively. The N‐Sensor, for instance, showed a linear relationship (Pearson's *r* = −0.99) between OCP and nitrate concentrations, with a sensitivity down to 1 ppm. Further, the Ca‐Sensor displayed a linear response to calcium concentrations ranging from 20 to 400 ppm, achieving a Pearson's r value of 0.99. Similarly, the P‐Sensor demonstrated strong responsiveness to hydrogen phosphate ions with a high coefficient of determination (Pearson's r = −0.98). Selectivity studies confirmed the sensors' ability to distinguish target ions from interferents such as potassium, sodium, and chloride. The Ca‐Sensor exhibited an RSD of %6.04, the N‐Sensor %4.50, and the P‐Sensor %9.10 under challenging conditions, indicating their good selectivity. These results indicate the sensors' reliability and suitability for precise ion detection, facilitated by their specific ion‐selective membranes and hydrophobic POT polymer layers that minimize interference from nontarget ions. We noted that the solid‐contact POT layer, recognized for its outstanding redox and hydrophobic properties, serves as a highly effective ion‐to‐electron transducer.^[^ ^55^ ^]^ It has been successfully employed in a variety of applications, including the detection of heparin,^[^ ^56^ ^]^ potassium,^[^ ^55a^ ^]^ iodide,^[^ ^55a^ ^]^ soil nitrate,^[^ ^57^ ^]^ and perchlorate^[^ ^58^ ^]^ in different liquid mediums.

The stability of the sensor was significantly enhanced by incorporating a thin protonated Nafion layer onto the reference electrode, as illustrated in Figure 1. This addition effectively prevented chloride ion leaching from the reference electrode throughout the measurement period. Remarkably, this enhanced stability enabled the sensor to maintain consistent long‐term sensing performance, with minimal deviation in the output signal observed after 45 h of continuous measurements. These findings underscore the electrode's robust resilience even under prolonged exposure to high concentrations of chloride ions, confirming its reliability for continuous sensing applications.

Integrating microfluidic systems with sensors is crucial because it enables precise, real‐time monitoring and analysis of small volumes of fluids. We successfully integrated a microfluidic system featuring a serpentine microchannel with dimensions of 500 µm in diameter and 171 mm in length. This setup effectively facilitates the rapid equilibration of milk samples, ensuring they reach ambient temperature efficiently. During testing, we utilized a peristaltic pump (300 µL min^−1^) to drive the sample through the microchannel, which was integrated with a Ca‐sensor. This approach not only demonstrates the system's capability to handle complex fluid dynamics but also validates its potential for precise, real‐time monitoring of calcium levels in milk samples. This integration marks a significant step forward in developing a robust platform for sensitive and portable dairy quality assessment applications. Finally, we designed a robust, pocket‐size, field‐deployable system for unsupervised use in advancing our sensor's technology for real‐world applications. The developed device integrates any of the four sensors and the microfluidic channel within a 3D‐printed enclosure, ensuring mechanical stability and ease of handling. This design not only protects delicate components from environmental factors but also facilitates seamless integration with a peristaltic pump for precise fluid manipulation. Figure 5D illustrates the CAD‐rendered schematic, showcasing how the sensor interfaces with the microchannel, optimizing detection efficiency. Such advancements are poised to revolutionize on‐site analysis, particularly in scenarios like milk sample analysis where reliability and portability are paramount.

The SLA printer used in this study can only print with specific UV‐curable inks and produces microgeometries, unlike e‐beam evaporation, which creates 2D flat surfaces. Our goal is to avoid lithography and instead focus on manufacturing low‐cost, 3D‐printed sensors that can be easily integrated into customized automated systems, such as milking machines used in dairy farms, and wastewater management systems.

In summary, this paper explores the application of stereolithography‐based extrusion printing to fabricate sensors using photopolymer resin, resulting in structures with unique wrinkled patterns. This additive manufacturing method enables the creation of sensors with intricate geometry and diverse material combinations, avoiding the need for costly cleanroom processes. Incorporating a universal solid‐contact POT layer, these sensors exhibit exceptional capabilities in multi‐ion sensing and selectivity. They offer rapid response times and enhanced stability, supported by a protonated Nafion layer that extends their performance longevity in multi‐ion detection. The hydrophobic nature of the solid‐contact POT enhances selectivity by repelling interfering ions, stabilizing sensor interfaces, and facilitating precise ion detection while minimizing background noise. The microfluidic system aims to counteract the negative effects of temperature fluctuations on accurate measurements, while our compact 3D‐printed device enables convenient on‐site application of our sensors. This advancement represents a significant stride in microelectronics fabrication for multi‐ion sensing, enabling detection even at low concentration ranges across various media. This proof‐of‐concept device successfully detects various ions, including Fe^2^⁺, Ca^2^⁺, HPO₄^2^⁻, and NO₃⁻, and we plan to integrate four sensing electrodes into a single device to enable simultaneous detection of all these ions in the future.

## 4 Experimental Section

### Chemicals

Deionized water with a resistivity of 18.2 MΩ cm was obtained using a Millipore purification system from the US. Chemicals including calcium chloride (CaCl_2_), sodium chloride (NaCl), sodium phosphate dibasic (Na_2_HPO_4_), potassium chloride (KCl), and Ag|AgCl ink (composed of finely dispersed chloridized silver flakes) were obtained from Fisher Scientific, Waltham, MA, USA. Polyvinyl chloride (PVC), POT, tetrahydrofuran (THF), 1,10‐phenanthroline, potassium tetrakis chlorophenyl borate (KTCpB), dibutylphthalate (DBP), sodium tetrakis[3,5‐bis(trifluoromethyl)phenyl] borate, dioctyl sebacate (DOS), tributyltin chloride, 2‐Nitrophenyl octyl ether (NPOE), methyltriphenylphosphonium bromide, tridodecylmethylammonium nitrate, tributyltin chloride, Sodium tetrakis[3,5‐bis(trifluoromethyl)phenyl]borate (NaTFPB), calcium ionophore II, and Nafion were purchased from Sigma–Aldrich (St. Louis, MO, USA). SYLGARD 184 PDMS Elastomer and curing agent purchased from Sigma–Aldrich. The high‐temperature resin cartridge (V2) used for manufacturing the sensor base was purchased from Dynamism, Inc., Chicago, IL.

### Sensor Manufacturing

Figure 1C displays photographs of the actual sensors alongside surface geometry views of the printed sensor. The entire sensor geometry was manufactured using an extrusion‐based stereolithography printing tool. Prior to printing, a detailed 3D model was designed using Autodesk Fusion software. Each sensor included one working electrode and a reference electrode, situated within a base measuring 33 mm in length, 25 mm in width, and 2 mm in height. The WE had a diameter of 4 mm, while the RE was 3 mm in diameter. Both electrodes and channels were 0.3 mm deep, with a 0.2 mm outer circle around each electrode designed to contain liquid samples. The sensor base was fabricated using a high‐temperature resistant resin from Formlabs, Inc., utilizing an extrusion‐based 3D printer. Ten sensors were successfully printed simultaneously. Post‐printing, each sensor underwent cleaning with isopropyl alcohol (IPA) using a FormWash L unit. Subsequently, the sensors were cured for 2 h in a FormCure L system at 80 °C under 405 nm UV light (Figure 1A). This curing process enabled the cross‐linking of epoxy resin molecules, forming a cohesive polymer network. The lateral layer width of ≈80 µm was determined by the laser source's precision used to cure the resin during the printing process. This width was created as the laser traced each layer's pattern on the resin surface, selectively solidifying specific regions. Factors such as the laser spot size or the projector's pixel size affect the lateral resolution, including the layer width. Adjustments to the lateral layer width can be made during fabrication by modifying parameters like the laser spot size, printing angle, or optics used to focus the beam. Additionally, settings such as exposure time and layer thickness also play a role in determining the precision of the layer width, though these adjustments are constrained by the printer's hardware capabilities.

Subsequently, a layer of gold was deposited on each electrode. This coating was accomplished by placing a shadow mask. The mask, made from Kapton tape cut to the 2D sensor design using an automated silhouette cutter, was applied. A 10 nm thick chromium (Cr) layer served as an adhesive, followed by the deposition of a 100 nm thick gold layer using an e‐beam evaporator. This gold layer provided conductivity on the wrinkled sensor surface.

For the modification of WE, a POT transducing layer solution was prepared by mixing POT with THF. This POT solution was drop‐casted (20 µL) onto the gold‐coated WE and allowed to dry overnight. For the reference electrode, a silver|silver chloride solution was applied over the gold layer and dried. Subsequently, a Nafion anti‐leaching layer was added to enhance the electrode's longevity. To complete the electrode assembly, copper conductive tape was affixed to the ends of the gold layer beneath the electrodes. Silver conductive paint was then used to secure the tape in place, preventing movement or detachment.

For the Fe–Sensor, the Fe–ISM cocktail solution was prepared using a mixture of KTCpB, DBP, and PVC in proportions of %6.73, %3.33, %60, and %30, respectively, along with THF.^[^ ^59^ ^]^ A 20 µL of this solution of the Fe‐ISM was then added on top of the POT layer and dried overnight. For the Ca–Sensor, a Ca‐ISM cocktail solution was prepared with %1 Ca^2+^ selective ligand, %65 plasticizer, %1 additive, and 33% polyvinyl chloride powder.^[^ ^60^ ^]^ A 20 µL solution of this cocktail was drop‐casted onto the POT layer. For the N–Sensor, an N−ISM cocktail contained methyltriphenylphosphonium bromide (%0.25), nitrocellulose (moistened with 2‐propanol, %1.93), 2‐nitrophenyl octyl ether (%16.25), polyvinyl chloride (%5.75), THF (%74.3), and tridodecylmethylammonium nitrate (%1.50).^[^ ^40b^ ^]^ It was sealed and stored at −20 °C before use. For the P‐sensor, the P–ISM cocktail was prepared using 3.0 mg (1% wt) of tributyltin chloride, 198 mg (66% wt) of NPOE, 99 mg (33 wt%) of PVC, and 2.0 mg of NaTFPB in 3 mL of THF.^[^ ^61^ ^]^ A 20 µL of this cocktail will be coated on the POT surface to create the P–Sensor. All four sensors were conditioned by immersing them in highly concentrated (3 m) solutions of Fe^2+^, NO_3_^–^, Ca^2+^, and HPO_4_^2−^ for over 12 h. This step aimed to replace interfering ions in the membrane with desired ions, ensuring reliable sensor performance. Conditioning was performed before calibration and selectivity tests, to achieve stable and accurate responses.

The PDMS elastomer was mixed with its curing agent in a ratio of 10 parts elastomer to 1 part curing agent by weight. After degassing in a vacuum desiccator, the mixture was poured onto the master mold placed in a petri dish, and allowed to sit at ambient temperature for 24 h. Subsequently, the master mold was removed from the PDMS negative replica, which was then washed several times with IPA and DI water.

### Electrochemical Characterization Tests

Electrochemical characterization techniques cyclic voltammetry (CV), differential pulse voltammetry (DPV), and electrochemical impedance spectroscopy (EIS) using ferro/ferricyanide in KCl solution were conducted. Given that the formation constant (K_f_) of the gold‐cyanide complex ([Au(CN)2]⁻) is ≈10^40^ m ^−^^2^, which is notably high, a specific protocol was followed.^[^ ^62^ ^]^ This included using a fresh gold electrode for CV, DPV, and EIS analysis, minimizing exposure time, and cleaning the electrode afterward to remove any adsorbed cyanide. It was noted that the electrode used for electrochemical characterizations was not used for sensor fabrication.

### Statistical Analysis

Statistical analysis was performed with OriginPro 2023b and GraphPad Prism 10 software (GraphPad Software, Inc.), and all data are presented as mean ± SD. The results are represented visually using error bars and shaded error regions in the graphs. Independent experiments were conducted at least three times (*n* = 3) unless stated otherwise. For all calibration graphs and the temperature correlation graph, linear regression analyses were conducted using OriginPro 2023b software, and Pearson's r values were calculated and included with each calibration graph. The relative standard deviation (RSD) percentage was utilized to assess the repeatability and evaluate the interference influence in the study.

### Software

The following software tools were utilized for various aspects of the study: OriginPro 2023b and GraphPad Prism 10 for statistical analysis, Autodesk Fusion 2024 and AutoCAD 2024 for designing the 3D models used in 3D printing, and Biorender 2023 for creating schematics. Additionally, software associated with the extrusion SLA printer, equipped with different control managers, was employed to manage the photopolymer resin 3D printing process of the sensor base, device, and master mold.

## Conflict of Interest

The authors declare no conflict of interest.

## Supporting information



Supporting Information

## Data Availability

The data that support the findings of this study are available from the corresponding author upon reasonable request.
