# Supplementary material for: Advancing Multi‐Ion Sensing with Poly‐Octylthiophene: 3D‐Printed Milker‐Implantable Microfluidic Device
Source: Adv Sci (Weinh). 2024 Oct 14;11(47):2408314. doi: 10.1002/advs.202408314 (PMC11653713; doi:10.1002/advs.202408314)
Supplement: Supplementary file 1 — Supporting Information [file ADVS-11-2408314-s001.pdf]

ADVANCED SCIENCE
Open Access

Supporting Information

for *Adv. Sci.*, DOI 10.1002/advs.202408314

Advancing Multi-Ion Sensing with Poly-Octylthiophene: 3D-Printed Milker-Implantable Microfluidic Device

Md. Azahar Ali and Matin Ataei Kachouei*

Supporting Information

Advancing Multi-Ion Sensing with Poly-octylthiophene: 3D Printed Milker-Implantable Microfluidic Device

Md. Azahar Ali[1,2,*], and Matin Ataei Kachouei[1]

[1]School of Animal Sciences, Virginia Tech, Blacksburg, Virginia 24061, USA
[2]Biological Systems Engineering, Virginia Tech, Blacksburg, Virginia 24061, USA

Corresponding Author: Md. Azahar Ali; Email: azahar@vt.edu; Phone: +1–540–231–5253

Table of Content

S1. 2D and 3D Electrode Performance

S2. Electrochemical characterization of POT layer

S3. POT as hydrophobic transducer

S4. Repeatability study

S5. Temperature fluctuation effect

S6. Heat dissipation in microfluidic system

S7. pH effect on ion sensing

S8. 3D electrode profilometry

S1. 2D and 3D electrode performance

The increased surface area of the 2D and 3D electrodes is attributed to the periodic patterns formed during the sensor's printing process, which significantly enhances the surface area. This increase, in turn, expands the area of the POT layer, which functions as the transducing agent, thereby improving sensor performance by enhancing its sensitivity to small ion concentration changes in equilibrium with the ion-selective membrane.

To empirically demonstrate that the 3D-printed gold electrode with periodic patterns possesses a higher surface area compared to a 2D gold electrode of the same diameter, we conducted cyclic voltammetry (CV) and differential pulse voltammetry (DPV) in a solution containing 5 mM $[Fe(CN)_6]^{4-/3-}$ as the mediator and 0.1 M KCl as the supporting electrolyte. As shown in **Fig. S1A**, both oxidative and reductive peak currents for the 3D patterned electrode are three times greater than those observed for the 2D smooth-surfaced electrode. The peak current in CV is directly proportional to the electroactive surface area of the electrode, as described by the *Randles-Sevcik* equation (**eq. S1**)[1]:

$$I_P = (2.69 \times 10^5)\, n^{3/2} A D^{1/2} C v^{1/2} \qquad \textbf{eq. S1}$$

This observation confirms that, despite the electrodes having identical dimensions, the 3D gold electrode has a larger surface area than its 2D counterpart, resulting in greater electron consumption and generation during the oxidation and reduction of the mediator. Additionally, we employed DPV to further highlight the surface area differences between the two electrodes. The ΔI_{max} in DPV is directly related to the electroactive surface area (A) of the electrode, according to the equation (**eq. S2**)[1]:

$$\Delta I_{max} = \frac{nFA\sqrt{D}C}{\sqrt{\pi}(\tau - \tau')} \cdot \left(\frac{1-\sigma}{1+\sigma}\right) \qquad \textbf{eq. S2}$$

Since ΔI_{max} is directly proportional to the electrode's surface area, the 3D patterned gold electrode exhibits a larger electroactive surface area, as illustrated in **Fig. S1B**. These results underscore a three times higher surface area of the 3D electrode, which enhances the sensor's limit of detection (LOD) due to the increased area of the coated transducer.

These findings demonstrate that the surface area of the 3D electrode expanded threefold compared to the 2D electrode, owing to the presence of periodic and wrinkled patterns.

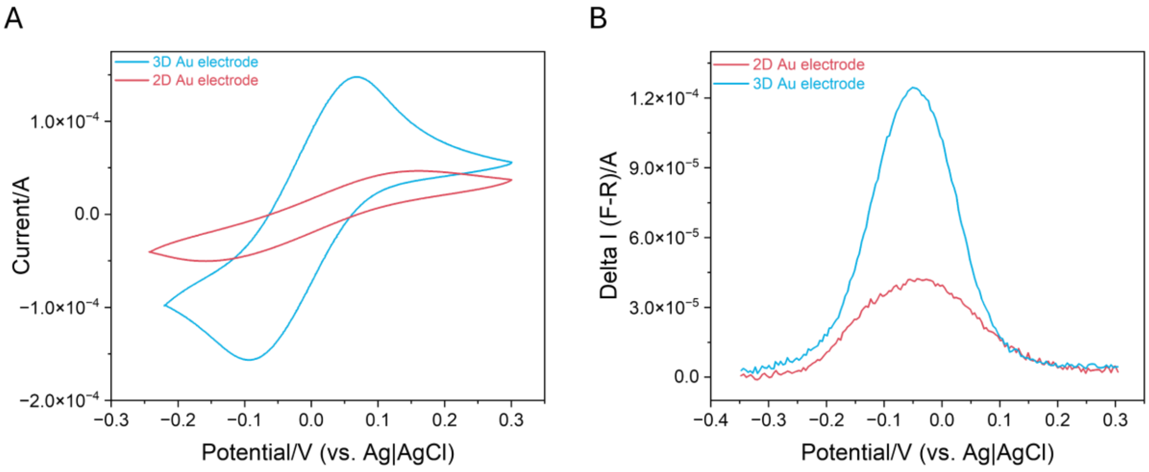

Figure S1. Sensor performance with 3D and 2D geometries. (A) Cyclic voltammograms and **(B)** differential pulse voltammograms of the 2D and 3D patterned gold electrodes recorded in a solution containing 5 mM $[Fe(CN)_6]^{4-/3-}$ and 0.1 M KCl, at a scan rate of 50 mV/s.

S2. Electrochemical characterization of POT layer

We performed Electrochemical Impedance Spectroscopy (EIS) analysis for a more comprehensive characterization of the surface electrochemical properties of the POT-modified electrode. Accordingly, we conducted EIS analysis on both the bare 3D gold electrode and the POT-coated gold electrode. The experiments were performed using a 5 mM $[Fe(CN)_6]^{4-/3-}$ electrolyte solution with 0.1 M KCl, over a frequency range of 100 kHz to 1 Hz and with an amplitude of 10 mV. The resulting Nyquist plots are presented in **Fig. S2**. Upon drop casting the POT ink onto the gold electrode, the semicircle diameter, corresponding to the charge transfer resistance (Rct), increased significantly compared to that of the bare gold electrode. Specifically, the Rct value for the bare gold electrode was determined to be 233.6 Ω, while the POT-coated gold electrode exhibited an Rct of 4808.3 Ω. This marked increase in Rct indicates a substantial decrease in the electrocatalytic activity of the gold electrode following the application of the POT layer. It is noteworthy that POT, as a conductive polymer, functions as an *ion-to-electron* transducer.

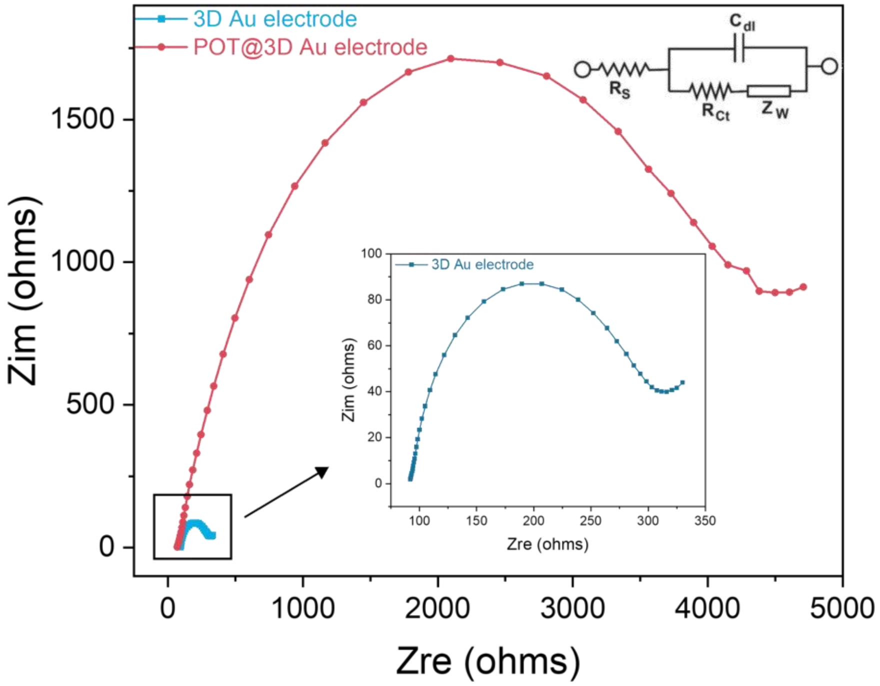

Figure S2. EIS analysis of the sensor. The Nyquist plot for the 3D Au electrode (blue) and the POT-coated 3D Au electrode (red), alongside the equivalent circuit model for the electrochemical system. The inset provides an enlarged view of the Nyquist plot for the 3D Au electrode. The electrolyte used in the experiment consists of 5 mM $[Fe(CN)_6]^{4-/3-}$ and 0.1 M KCl, with measurements conducted over a frequency range of 100 kHz to 1 Hz at an amplitude of 10 mV.

S3. POT as hydrophobic transducer

The presence of the POT layer in the ion sensor not only functions as the *ion-to-electron* transducer but also enhances the sensor's stability and minimizes potential drift due to its hydrophobic properties.[2] This hydrophobicity helps prevent water and other solutes from penetrating the space between the gold electrode surface and the ion-selective membrane (ISM). Therefore, we chose POT to serve as a hydrophobic transducer. The hydrophobic nature of the POT layer was confirmed by contact angle measurements, as shown in **Fig. S5A**. In this test, we coated a glass surface with POT and placed a 15 µL droplet of deionized water, resulting in a contact angle of 72°, demonstrating the hydrophobicity of the POT layer.

Moreover, to evaluate the transduction role of POT, we fabricated a calcium sensor without the POT layer and conducted open-circuit potential (OCP) analysis in standard Ca^{2+} solutions of 15, 350, 450, 550, and 700 ppm. As seen in **Fig. S5B**, the sensor lost its sensitivity in the absence of POT, as the transducer layer was no longer present.

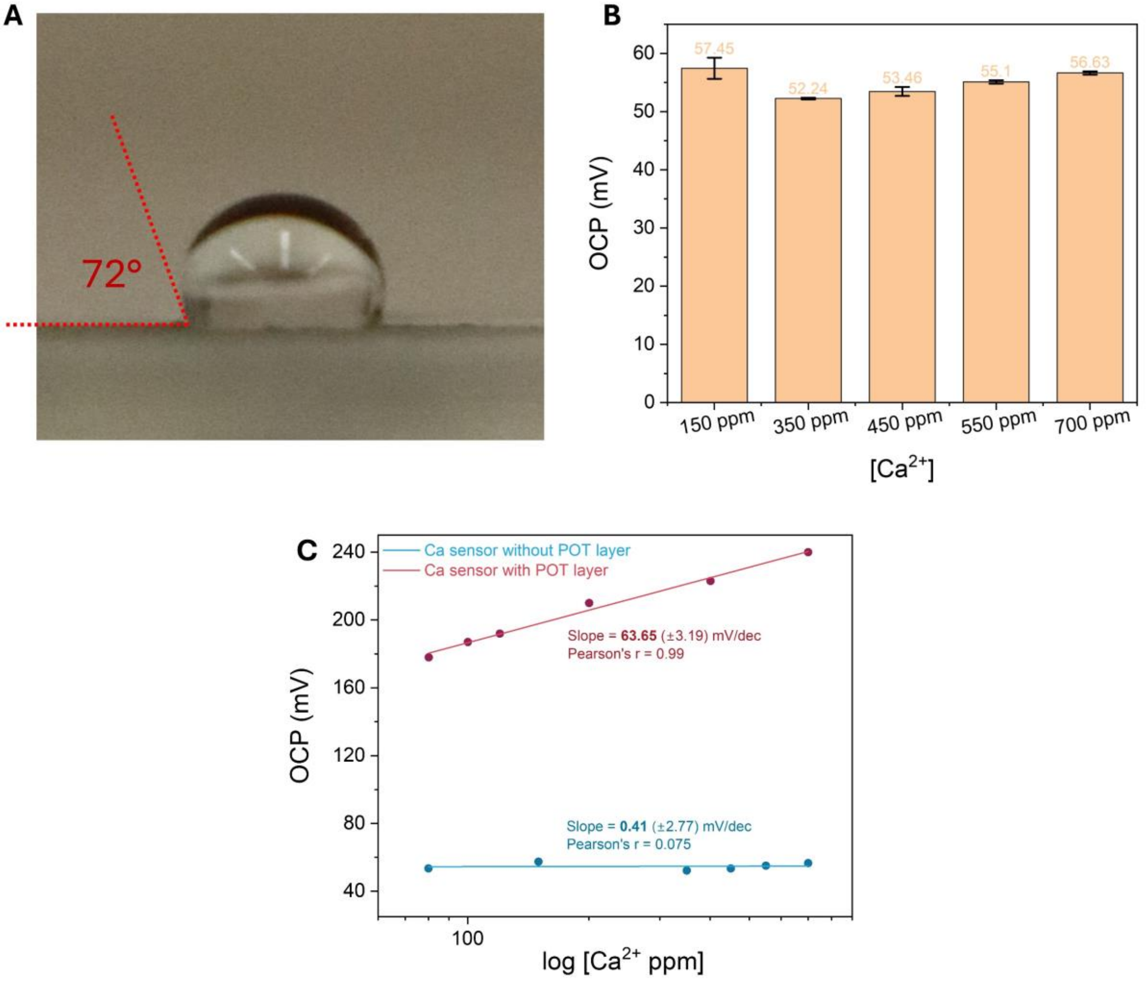

Figure S3. (A) Contact angle analysis of a 15 μL droplet of DI water on a POT-coated glass surface, conducted at room temperature and under atmospheric pressure. **(B)** The sensing response of the Ca sensor fabricated without POT layer toward various concentrations of Ca^{2+} ion. Each test was assessed by calculating the standard deviation based on three independent measurements (n=3) and reflected with error bars. **(C)** A comparison plot showing the slopes of the Ca-sensor calibration with and without POT layer.

S4. Repeatability study

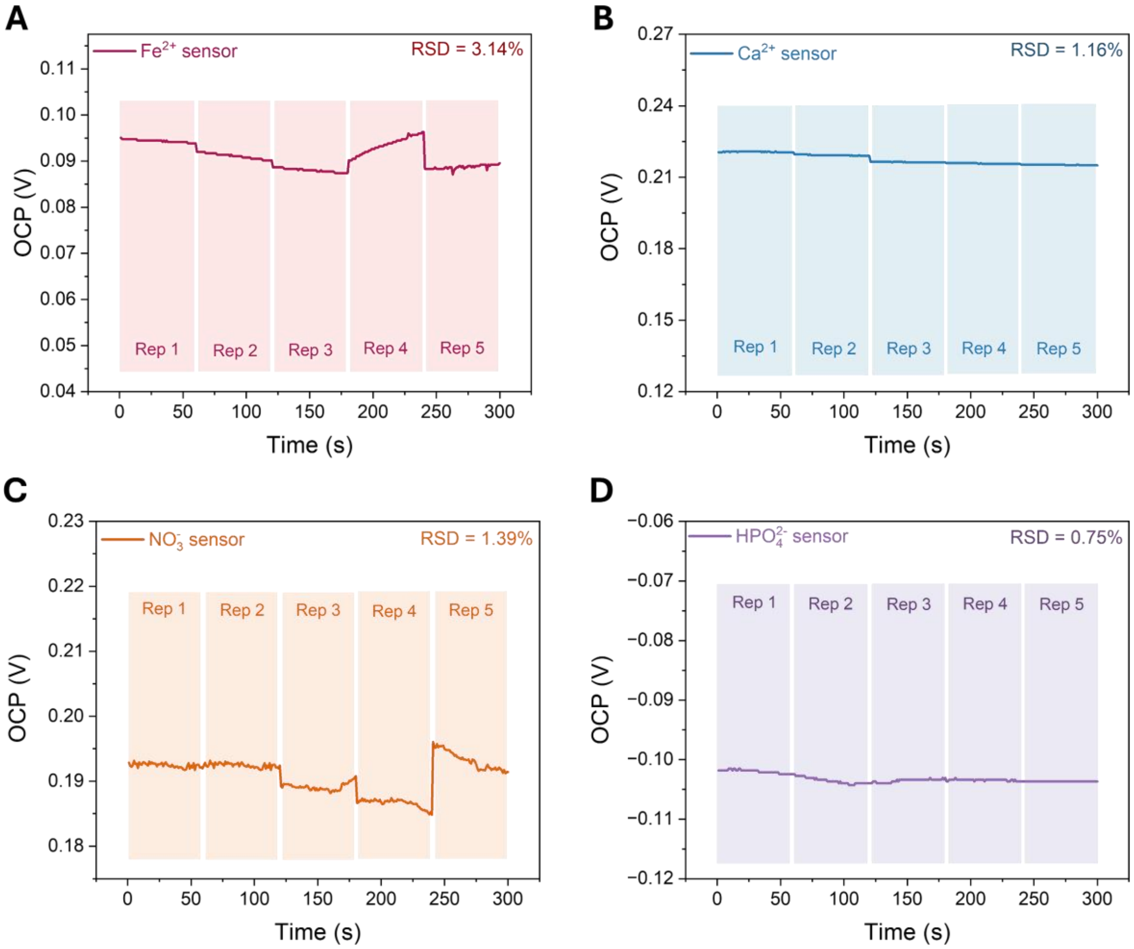

Figure S4. Repeatability analysis of sensors. (A) Five separate analyses were performed using the F sensor with a 1 ppm Fe^{2+} standard solution. Each of the five measurements was repeated three times (n = 3) under clinically independent conditions. The resulting relative standard deviation (RSD) was calculated to be 3.14%. **(B)** Five separate analyses were performed using the C sensor with a 200 ppm Ca^{2+} standard solution. Each of the five measurements was repeated three times (n = 3) under clinically independent conditions. The resulting relative standard deviation (RSD) was calculated to be 1.16%. **(C)** Five separate analyses were performed using the N sensor with a 500 ppm NO_3^- standard solution. Each of the five measurements was repeated three times (n = 3) under clinically independent conditions. The resulting relative standard deviation (RSD) was calculated to be 1.39%. **(D)** Five separate analyses were performed using the P sensor with a 100 ppm HPO_4^{2-} standard solution. Each of the five measurements was repeated three times (n = 3) under clinically independent conditions. The resulting relative standard deviation (RSD) was calculated to be 0.75%.

S5. Temperature fluctuation effect

To assess the potential impact of temperature fluctuations on the accuracy and precision of the sensor, we conducted a verification test using the Ca-sensor (as an example) with three standard solutions of 40 ppm Ca^{2+} at varying temperatures. In this experiment, the sensor was tested independently of the microfluidic system, as temperature variation is mitigated within that system. Specifically, one solution was maintained

at room temperature (25.0 °C ±0.1), another was heated to 45.0 °C ±0.1, and the third was cooled to 10.0 °C ±0.1, with all solutions having identical Ca^{2+} concentrations. We applied 300 µL of each solution to the sensor's sample holder and conducted open circuit potentiometry for 60 seconds. To minimize temperature fluctuations, the sensor was housed in a thermally isolated flask pre-conditioned to the target temperatures. As illustrated in **Fig. S5A**, temperature significantly influences measurement accuracy by altering the potential difference between the working and reference electrodes. At room temperature, where most sensors are typically calibrated, the OCP value was 157.3 mV. When the solution was cooled, the OCP decreased to 117.5 mV, while warming the solution caused the OCP to increase to 188.3 mV. According to the calibration equation for the calcium sensor (OCP = 70.01 log [Ca^{2+}] + 43.44), the sensor reading at room temperature was 41.9 ppm, closely matching the actual solution concentration. However, at lower and higher temperatures, the readings were 117.5 ppm and 11.4 ppm, respectively, clearly demonstrating the effect of temperature on the accuracy of potentiometric sensors. This outcome is attributed to the temperature dependence of the Nernst equation, which governs the relationship between ion activity (a) and electrode potential (E). As shown in **eq. 1** (main manuscript), an increase in temperature (T) leads to a reduction in the electrode potential (E), thereby directly affecting the overall cell potential.

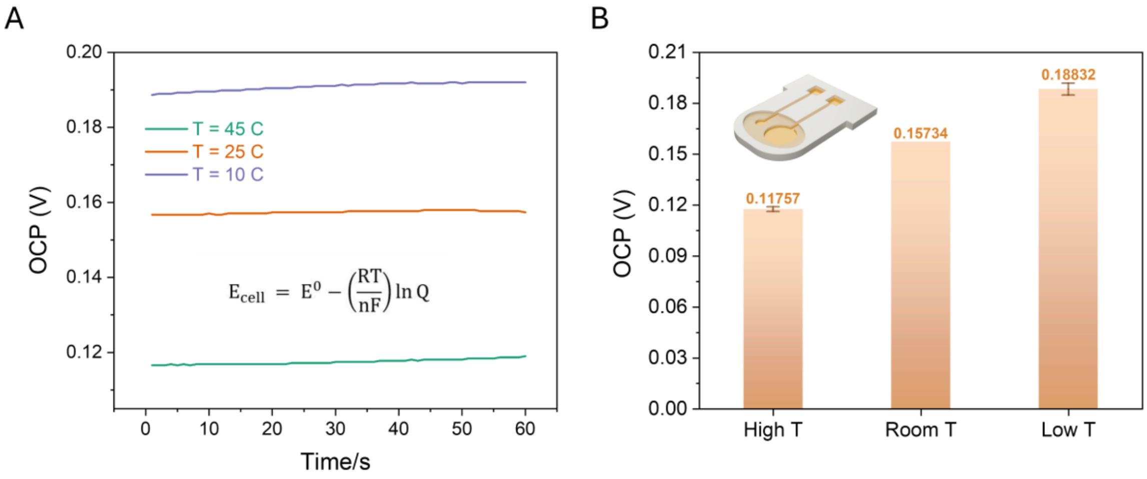

Figure S5. Temperature Influence on Ion Measurement. (A) Open Circuit Potential (OCP) analysis of a Ca-sensor in a 40 ppm Ca^{2+} solution at three distinct temperatures. **(B)** OCP values with corresponding error bars for each measurement across the three temperature conditions.

To assess the impact of temperature variations on measurement precision, we conducted each of the aforementioned experiments three times (n = 3) and calculated the standard deviation (±SD), as represented by the error bars in **Fig. S5B**. The measurements taken at room temperature exhibited minimal SD across the three independent tests, whereas those conducted at extreme temperatures demonstrated higher SD, highlighting the influence of temperature fluctuations on ion concentration measurement accuracy. Consequently, we opted to design and fabricate a microfluidic system with 500 µm channel diameters, primarily to enhance heat dissipation, thereby improving the accuracy and precision of various sensors, including Ca-sensor, P-sensor, F-sensor, and N-sensor.

S6. Heat dissipation in microfluidic system

The purpose of the designed microfluidic system is to bring the sample's temperature down to ambient levels, particularly in scenarios where the sample temperature exceeds the ambient temperature. For example, when our device is integrated with a milking machine to measure calcium ion levels in milk, the freshly collected milk is approximately 39 °C. This temperature discrepancy between the sample and the calibration standards can lead to significant errors in ion concentration measurements. Therefore, the goal is to align the sample temperature with the ambient temperature at which the sensor is calibrated. In this context, one of the key applications of the microfluidic system is to regulate the sample's temperature, by increasing the surface area to volume ration. This high surface area enhances heat exchange between the fluid and the channel walls, leading to more efficient cooling.

To assess the efficiency of the microfluidic system in cooling the fluid (deionized water, DI), we set up the system with two digital resistance temperature detectors (RTDs) of the Pt100 probe type to measure the fluid temperature at the inlet and outlet. A micro peristaltic pump, operating at a flow rate of 300 µL/min, was used. This configuration is depicted in **Fig. S6B**. Additionally, **Fig. S6A** illustrates the bullet probe positioned near the outlet of the microtube for precise temperature measurement. A hotplate was employed to adjust the temperature of the inlet fluid. As shown in **Figs. S6D**, **E**, **G**, and **H**, and summarized in **Table S1**, the microfluidic system effectively lowers the fluid temperature. **Fig. S6C** presents the regression equation between the inlet and outlet temperatures, demonstrating linearity across the tested temperature range. Moreover, the bar graph in **Fig. S6F** indicates that the system is more effective at reducing the inlet fluid temperature when the initial temperature is higher.

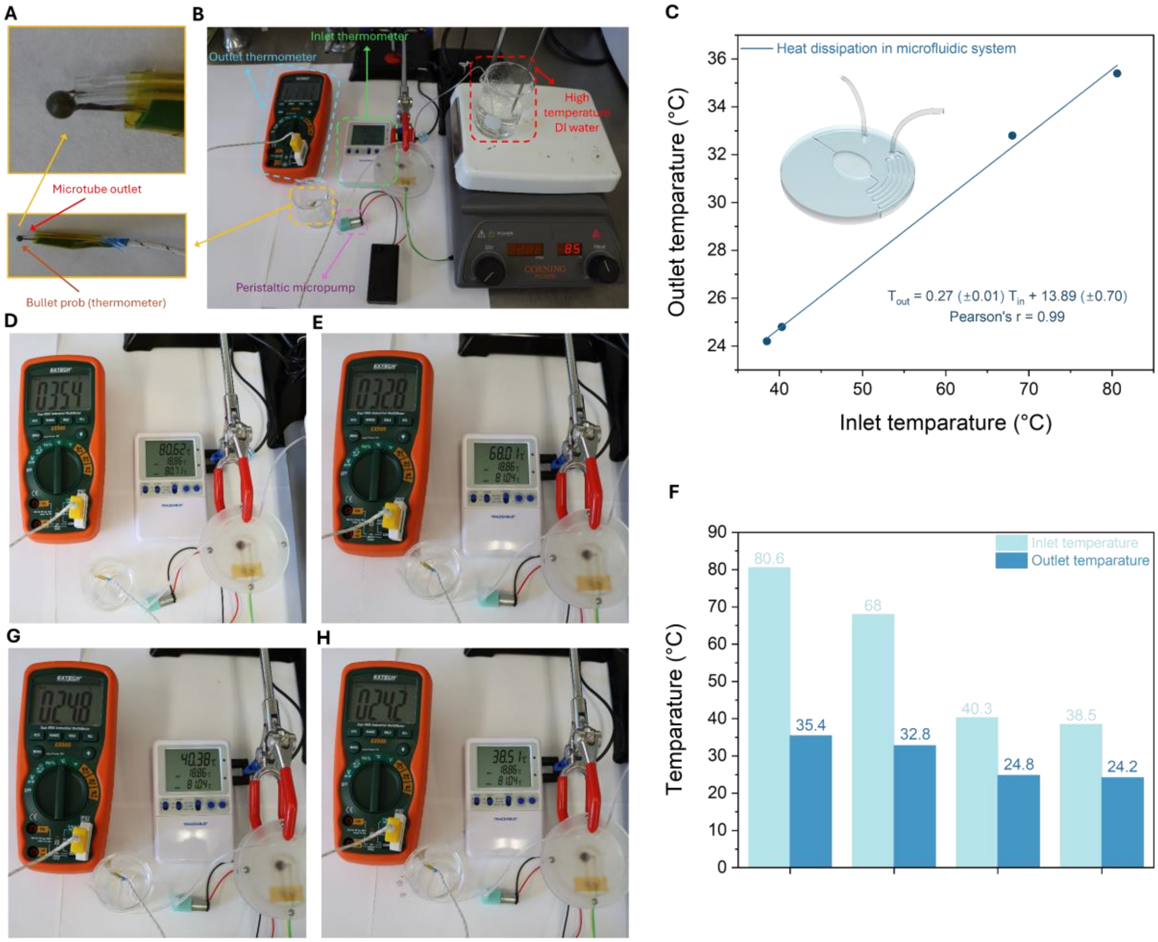

Figure S6. Microfluidic system for heat dissipation. (A) The placement of the bullet probe from the outlet RTD digital thermometer just at the exit of the outlet microtube. **(B)** The experimental design and setup for evaluating the heat dissipation efficiency of the microfluidic system. The inlet fluid (DI) is placed on a hotplate, with an RTD probe immersed in the fluid to monitor its temperature, while another RTD measures the outlet fluid temperature. A micro peristaltic pump drives the fluid at a flow rate of 300 µL/min. **(C)** The correlation graph between inlet and outlet temperatures, including the linear regression equation. **(D)** The inlet temperature is 80.6°C and the outlet temperature is 35.4°C. **(E)** The inlet temperature is 68.0°C and the outlet temperature is 32.8°C. **(F)** A bar graph displaying the inlet and outlet temperatures of the fluid for each experiment. **(G)** The inlet temperature is 40.3°C and the outlet temperature is 24.8°C. **(H)** The inlet temperature is 38.5°C and the outlet temperature is 24.2°C.

Table S1. The fluid's temperature at the inlet and outlet before and after passing through the microfluidic system.

	Inlet temperature (°C)	Outlet temperature (°C)	Flow rate (µL/min)
1	80.6	35.4	300
2	68.0	32.8	300
3	40.3	24.8	300
4	38.5	24.2	300

S7. pH effect on ion sensing

We have also assessed the impact of pH on the sensing performance of Fe and Ca sensors, as depicted in **Figs. S7A** and **S7B**, respectively. Although pH does not significantly affect sensing performance, it is important to calibrate sensors with samples in a medium as close as possible to their intended application.

The selectivity and pH dependency for the P sensor is different from the other sensors. The complexity of phosphate ion composition arises from hydrogen phosphate being a polyprotic acid. The solution's phosphate ion composition depends on the pH, as phosphate exists in various forms: H_3PO_4 (phosphoric acid), $H_2PO_4^-$ (dihydrogen phosphate), HPO_4^{2-} (hydrogen phosphate), and PO_4^{3-} (phosphate ion). The *Henderson-Hasselbalch* equation (**eq. S1**) describes the pH-dependent ratios of these species. Phosphoric acid has three dissociation steps with pKa values of approximately 2.1, 7.2, and 12.3, which define the pH ranges where each phosphate species predominates. Using this equation, we can analyze how pH affects the relative concentrations of each species: at low pH (below pKa_1), H_3PO_4 is dominant; near pKa_1, $H_2PO_4^-$ becomes more prevalent; around pKa_2, HPO_4^{2-} increases; and near pKa_3, PO_4^{3-} dominates in more basic conditions.

$$pH = pk_a + \log\left(\frac{[base]}{[Acid]}\right) \qquad \textbf{eq. S4}$$

Fig. S7D illustrates the fractional composition of phosphate ions (α) as a function of pH, calculated using the *Henderson-Hasselbalch* equation. The graph shows that HPO_4^{2-} is present in the pH range of 5 to 14, with its concentration varying based on other phosphate ions and solution pH. To evaluate the selectivity of our sensor for HPO_4^{2-} and experimentally confirm the pH effect, we tested a 500 ppm HPO_4^{2-} solution across a pH range of 6.5 to 11.0. **Fig. S7C** demonstrates that the sensor's response follows a sigmoidal curve shape with pH changes, reflecting variations in HPO_4^{2-}, $H_2PO_4^-$, and PO_4^{3-} concentrations. The pH range used in our experiments is highlighted in the fractional composition graph (**Fig. S7D**).

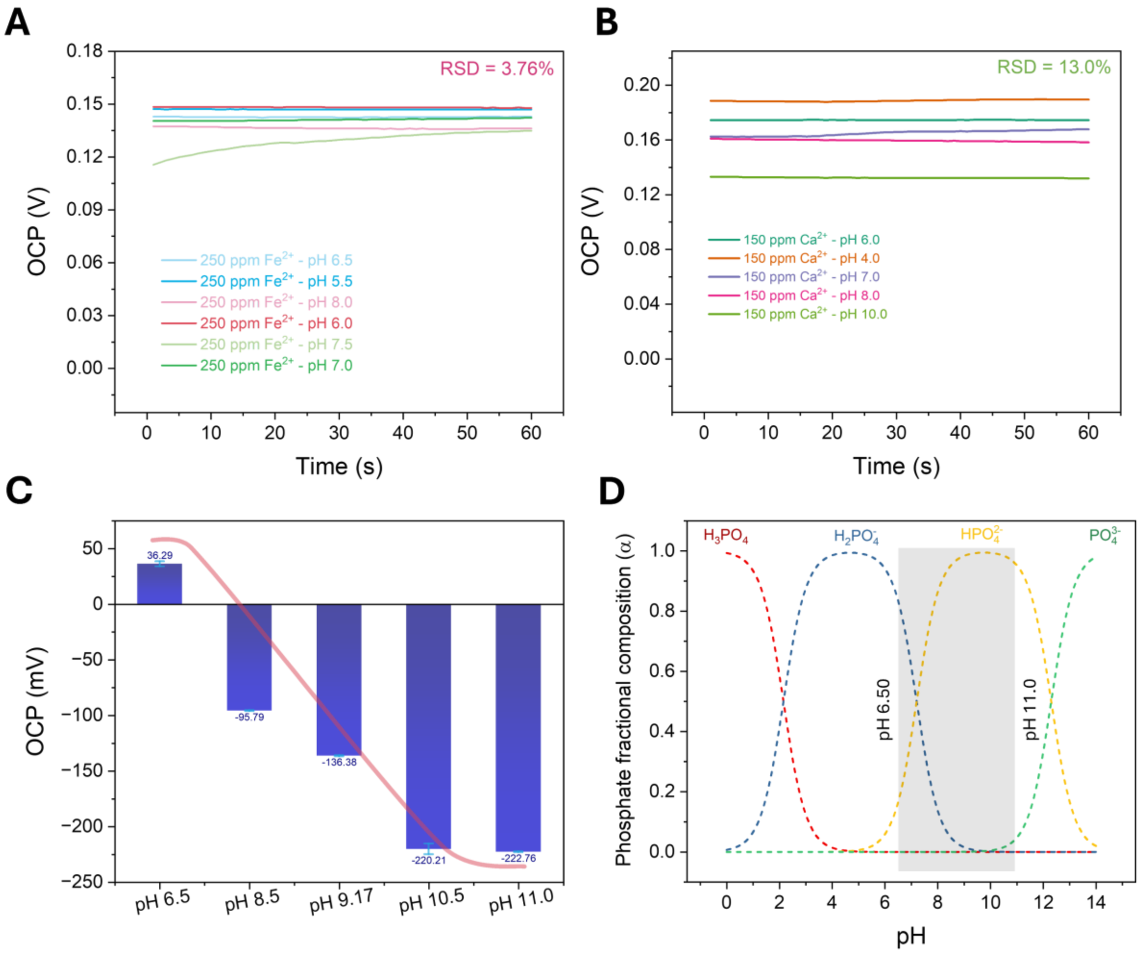

Figure S7. Selectivity and pH impact. (A) Potentiogram illustrating the pH effect on the sensing performance of the F sensor, with a relative standard deviation (RSD) of 3.76%. **(B)** Potentiogram depicting the pH effect on the C sensor's performance, with a relative standard deviation (RSD) of 13.0%. **(C)** Potentiogram showing P sensor performance in a 500 ppm HPO_4^{2-} solution under varying pH conditions. The precision of each sensor in sensing was assessed by calculating the standard deviation (SD) based on three independent measurements (n=3). The results are represented visually using error bars. **(D)** Fractional composition of phosphate at different pH values.

S8. 3D electrode profilometry

To provide height data of the polymer pattern, we performed surface profilometry on the electrode surface featuring periodic microstructures. We measured the surface profile by scanning the stylus perpendicular to the periodic microstructure patterns over a one-millimeter section of the electrode. The resulting profile, shown in **Fig. R2**, illustrates the periodic pattern peaks. The average peak height is 2.49 µm, with a standard deviation (SD) of 2.31 µm in printing the height. The variation in micropattern height is due to the profilometer measuring along a 2D line, where any random or wrinkled patterns can significantly affect the height. However, the SEM images show a consistent height for the micropatterns in a 3D viewpoint.

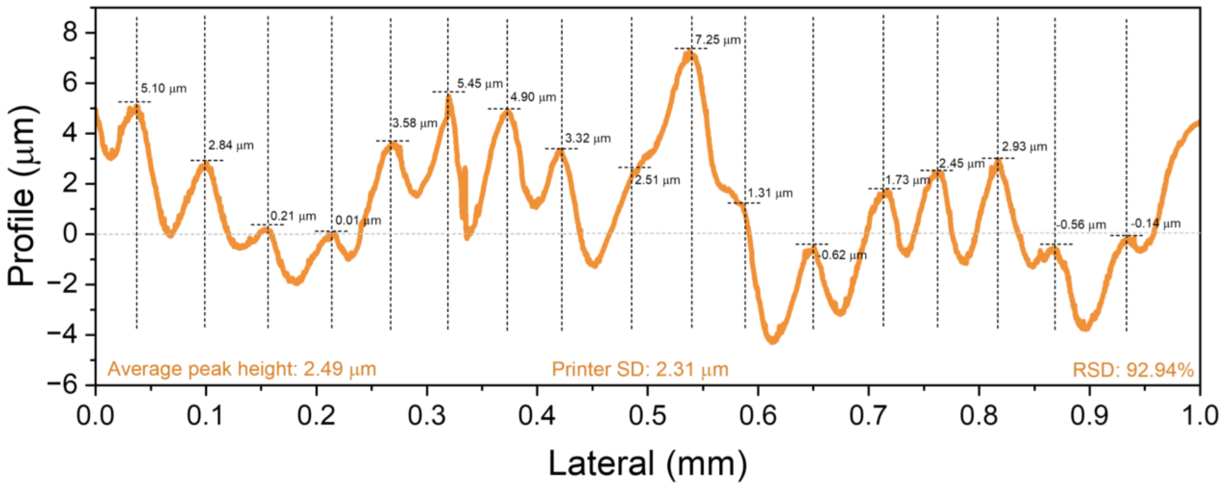

Figure S8. The profilometry measurements of the 3D-printed electrode surface revealed a periodic pattern. The stylus was oriented perpendicular to these patterns and scanned a 1-millimeter section of the electrode surface.

References:

1 Bard, A. J., Faulkner, L. R. & White, H. S. *Electrochemical Methods: Fundamentals and Applications*. (Wiley, 2022).

2 Jarvis, J. M., Guzinski, M., Pendley, B. D. & Lindner, E. Poly(3-octylthiophene) as solid contact for ion-selective electrodes: contradictions and possibilities. *Journal of Solid State Electrochemistry* **20**, 3033-3041 (2016). https://doi.org:10.1007/s10008-016-3340-2
